# Molecular basis of *A. thaliana* KEOPS complex in biosynthesizing tRNA t^6^A

**DOI:** 10.1093/nar/gkae179

**Published:** 2024-03-13

**Authors:** Xinxing Zheng, Chenchen Su, Lei Duan, Mengqi Jin, Yongtao Sun, Li Zhu, Wenhua Zhang

**Affiliations:** School of Life Sciences, Key Laboratory of Cell Activities and Stress Adaptation of the Ministry of Education, Lanzhou University, Lanzhou 730000, China; School of Life Sciences, Key Laboratory of Cell Activities and Stress Adaptation of the Ministry of Education, Lanzhou University, Lanzhou 730000, China; School of Life Sciences, Key Laboratory of Cell Activities and Stress Adaptation of the Ministry of Education, Lanzhou University, Lanzhou 730000, China; School of Life Sciences, Key Laboratory of Cell Activities and Stress Adaptation of the Ministry of Education, Lanzhou University, Lanzhou 730000, China; School of Life Sciences, Key Laboratory of Cell Activities and Stress Adaptation of the Ministry of Education, Lanzhou University, Lanzhou 730000, China; School of Life Sciences, Key Laboratory of Cell Activities and Stress Adaptation of the Ministry of Education, Lanzhou University, Lanzhou 730000, China; School of Life Sciences, Key Laboratory of Cell Activities and Stress Adaptation of the Ministry of Education, Lanzhou University, Lanzhou 730000, China

## Abstract

In archaea and eukaryotes, the evolutionarily conserved KEOPS is composed of four core subunits―Kae1, Bud32, Cgi121 and Pcc1, and a fifth Gon7/Pcc2 that is found in fungi and metazoa. KEOPS cooperates with Sua5/YRDC to catalyze the biosynthesis of tRNA *N^6^*-threonylcarbamoyladenosine (t^6^A), an essential modification needed for fitness of cellular organisms. Biochemical and structural characterizations of KEOPSs from archaea, yeast and humans have determined a t^6^A-catalytic role for Kae1 and auxiliary roles for other subunits. However, the precise molecular workings of KEOPSs still remain poorly understood. Here, we investigated the biochemical functions of *A. thaliana* KEOPS and determined a cryo-EM structure of *A. thaliana* KEOPS dimer. We show that *A. thaliana* KEOPS is composed of KAE1, BUD32, CGI121 and PCC1, which adopts a conserved overall arrangement. PCC1 dimerization leads to a KEOPS dimer that is needed for an active t^6^A-catalytic KEOPS–tRNA assembly. BUD32 participates in direct binding of tRNA to KEOPS and modulates the t^6^A-catalytic activity of KEOPS via its C-terminal tail and ATP to ADP hydrolysis. CGI121 promotes the binding of tRNA to KEOPS and potentiates the t^6^A-catalytic activity of KEOPS. These data and findings provide insights into mechanistic understanding of KEOPS machineries.

## Graphical Abstract

**Graphical Abstract:**
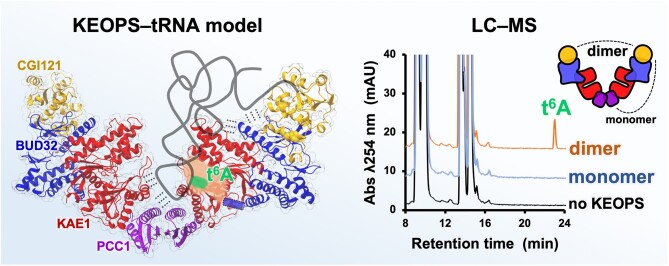

## Introduction

In *Saccharomyces cerevisiae*, Kae1, Bud32, Cgi121, Pcc1 and Gon7 associate to form a stable protein complex that was dubbed KEOPS (Kinase, Endopeptidase and Other Proteins of Small size) (1) or EKC (Endopeptidase-like Kinase Chromatin-associated) (2). Deletion of genes encoding KEOPS/EKC (KEOPS hereafter) subunits or mutations disrupting the integrity of KEOPS complex led to lethality of budding yeast (1,2), which also manifested a plethora of phenotypes that include shortened telomeres (1,3–6), accumulation of DNA double-strand breaks (DSBs) (7), transcriptional inactivation of pheromone- and galactose-responsive genes (2,5,8), and global translational dysregulation (9,10). Comparative genomics analysis revealed that KEOPS proteins are universally encoded in genomes of archaea and eukaryotes (11), with exception for Gon7/GON7 that is presently only found in yeast (1) or humans (12). No ortholog of Cgi121 is encoded in *Drosophila* genome (11,13,14). Knockouts of KEOPS genes severely affect growing and development of higher eukaryotes, such as reduced size of larval of fruit flies (14,15), microcephaly and early lethality of zebrafish and mice (16,17). In humans, pathogenic mutations of KEOPS subunits―OSGEP (Kae1), PRPK (Bud32), TPRKB (Cgi121), LAGE3 (Pcc1) and GON7 (Gon7)―are implicated in the heterogeneous autosomal recessive Galloway–Mowat syndrome (GAMOS) that is characterized by early-onset steroid-resistant nephrotic syndrome and microcephaly (16,18).

*In vitro* enzymatic reconstitution demonstrated that KEOPS participates in biosynthesis of tRNA *N^6^*-threonylcarbamoyladenosine (t^6^A) (12,19–21), which is an essential post-transcriptional modification needed for correct decoding and translational regulation (9,10,22–25). t^6^A belongs to a core set of 18 ‘universal’ post-transcriptional modifications that are found in tRNAs from three domains of life (26,27). t^6^A is uniquely installed at position 37 of tRNAs that decipher codons starting with adenine, namely, ANN codons (N being A, U, C or G) (26). In t^6^A-modified tRNAs, t^6^A extends its planar ring via intramolecular hydrogen bonds and π–π stacking interaction with U36 and prevents the intra-loop Watson–Crick pairing between U33 and A37 (28,29). Genetic and biochemical analysis revealed that the biosynthesis of tRNA t^6^A requires members from two last universal common ancestor protein families―TsaC/Sua5 (COG0009) (30) and TsaD/Kae1/Qri7 (COG0533) (10), which cooperate to carry out two consecutive reactions (11,31,32). In the first step, TsaC/Sua5 protein utilizes *L*-threonine, CO_2_/HCO_3_^−^ and ATP to generate an intermediate *L*-threonylcarbamoyladenylate (TC-AMP) (33,34); in the second step, TsaD/Kae1/Qri7 protein catalyzes the transfer of TC-moiety from TC-AMP onto N6 atom of tRNA A37, leading to tRNA t^6^A (34,35). However, such a two-component biosynthetic system only exists in eukaryotic mitochondria (34–36). In bacteria, the transfer of TC-moiety is catalyzed by TsaD, TsaB and TsaE (33,37,38), which form an ATP-mediated TsaD–TsaB–TsaE complex (TsaDBE hereafter) (38–42). In archaea and eukaryotes, the TC-transfer is catalyzed by Kae1/OSGEP in form of KEOPS complex (12,18,43–46). Interestingly, TsaC- and TsaD-like domains are fused in TsaN from *Pandoraviruses* and are capable of catalyzing *N*^6^-threonylcarbamoylation modification of adenosine di-/tri-phosphates at nucleotide level, leading to t^6^ADP and t^6^ATP (47). The structural similarity in TsaC/Sua5 proteins (47–51) or TsaD/Kae1/Qri7 proteins (18,39,41,47,52–54) implies a conserved catalytic mechanism underlying TC-AMP formation or TC-transfer reactions (32). However, biochemical analysis showed large variations in t^6^A modification efficiencies of different t^6^A-modifying enzymes towards same tRNA substrates, and *vice versa* (12,31,36,43,44,55). It appears that the TsaD/Kae1/Qri7 family proteins have evolved more auxiliary components to regulate t^6^A-modifcation frequencies of tRNAs in coping with increasing biological complexity (11,31,32).

Crystal structures of KEOPS subcomplexes allowed reconstruction of structural models for KEOPS from archaea (3,44), yeast (56) and humans (18,57). These three models of KEOPS showed that the core subunits adopt a conserved linear architecture depicted as Pcc1/LAGE3–Kae1/OSGEP–Bud32/PRPK–Cgi121/TPRKB (31,32). Gon7/GON7 is an intrinsically disordered protein and interacts solely with Pcc1/LAGE3 in KEOPS (18,56). Recently, a paralog of archaean Pcc1, dubbed Pcc2, interacts with Pcc1 in a manner that is analogous to Gon7/GON7 (46). Biochemical analysis demonstrated that the four-subunit KEOPS from archaea forms a dimer (20,44) whereas the five-subunit KEOPSs from yeast and humans cannot form a dimer due to the presence of Gon7/GON7 (12,18,56). The dimerization of Pcc1 leads to formation of archaean KEOPS dimer that is required for *in vitro* tRNA t^6^A biosynthesis (44,46). Subtraction of Pcc1 from archaean KEOPS leads to dead t^6^A-catalytic activity of the subcomplex Kae1–Bud32–Cgi121 (20). Deletion of Gon7/GON7 severely affects tRNA t^6^A biosynthesis by KEOPS in yeast and humans (12,18,34). Likewise, replacement of Pcc1 by Pcc2 in archaean KEOPS still sustains the dimeric state but leads to loss of t^6^A catalytic activity (46). Crystal structure of *M. jannaschii* (*Mj*) Cgi121–tRNA complex allowed generation of a structural model of archaean KEOPS–tRNA complex (45). According to this model, anticodon stem loop of tRNA is anchored in the concave formed between Pcc1 and Kae1, allowing tRNA A37 to protrude into the t^6^A-catalytic center of Kae1; D stem loop of tRNA simultaneously makes contacts with Kae1 and Bud32; 3′ CCA end is bound by Cgi121; TψC stem loop does not participate in direct interaction with KEOPS. This model delineates a plausible binding mode for KEOPS–tRNA and explains the roles of individual subunits (31,45). However, precise molecular interactions between KEOPS and tRNA are still not determined, e.g. the binding of tRNA A37 in catalytic site of Kae1 is still enigmatic. Nonetheless, a manual adjustment of tRNA crystal structure is needed to geometrically fit an extended surface of the four subunits of KEOPS (45), suggesting that binding of tRNA to KEOPS might mutually induce large conformational changes in structures of KEOPS and tRNA. Structural analysis and biochemical validations demonstrated that t^6^A-catalytic activity of KEOPS necessitates an ATP to ADP hydrolysis by Bud32 (19,20,45,56,57). At present, it's poorly understood as how KEOPS subunits cooperate to regulate KEOPS–tRNA assembly and t^6^A modification efficiency. Therefore, an atomic structure of a complete KEOPS complex is desirable to investigate the precise roles and molecular workings of KEOPS in tRNA t^6^A biosynthesis.

Comparative genomics analysis revealed that orthologs of the four core subunits of KEOPS―Kae1, Bud32, Cgi121 and Pcc1―are encoded in *Arabidopsis thaliana* genome (11). However, the biochemical functions and structures of *A. thaliana* KEOPS proteins have not been characterized. Moreover, liquid chromatography–mass spectrometry (LC–MS) analysis revealed t^6^A and its hypermodified derivative–*N*^6^-methyl t^6^A (m^6^t^6^A) in tRNAs isolated from *Arabidopsis thaliana* (58). Yet, the biochemical pathway of tRNA t^6^A biosynthesis remains uncharacterized. In this study, we reconstituted an enzymatic biosynthesis of tRNA t^6^A using purified recombinant proteins of *A. thaliana* KEOPS (KAE1, BUD32, CGI121 and PCC1) and YRDC. We reconstituted *A. thaliana* KEOPS complex and investigated molecular mechanisms of *A. thaliana* KEOPS in tRNA t^6^A biosynthesis. Here, we report the cryo-EM structure of *A. thaliana* KEOPS and structure–function relationship analysis of *A. thaliana* KEOPS–tRNA assembly, which extend our current mechanistic understandings of the ancient KEOPS machineries.

## Materials and methods

### Plasmid construction, protein expression and purification

We constructed expression plasmids of *Arabidopsis thaliana* (*At*) KEOPS proteins using chemically synthesized DNAs encoding *At*YRDC (Gene ID: 836180), *At*KAE1 (Gene ID: 828368), *At*BUD32 (Gene ID: 832 680), *At*CGI121 (Gene ID: 829592) and *At*PCC1 (Gene ID: 835384). In total, we constructed plasmids of pET21a–*At*YRDC-6His, pET26a–*At*KAE1–BUD32–CGI121–PCC1-6His, pET21a–*At*KAE1–BUD32-6His, pET26a–*At*KAE1–PCC1-6His, pJ241–*At*BUD32–CGI121-6His and pET21a–*At*CGI121-6His, which express six histidine (6His)-tagged YRDC, KEOPS, KAE1–BUD32, KAE1–PCC1, BUD32–CGI121 and CGI121, respectively. In addition, we constructed 30 plasmids that express *At*KEOPS variants with site-directed mutagenesis following the manufacturer's protocol (Takara). The primers are listed in Supplementary Table S1.

Expression plasmids of *At*KEOPS proteins were transformed into *Escherichia coli* (Rosetta DE3 pLysS or BL21) and cultured overnight at 37°C. The transformant preculture was inoculated 1/100 (v/v) in LB or 2YT medium and grown until the OD_600_ reached 0.5–0.8, induced with addition of 0.5 mM isopropyl β-d-1-thiogalactopyranoside (IPTG) for 3 hours at 37°C or for 20 hours at 16°C. *S. cerevisiae* (*Sc*) KEOPS and *P. salinus* (*Ps*) TsaN^1–392^ were expressed using plasmids pJ241–*Sc*KEOPS-6His (56) and pET24a–TsaN^1–392^ (47). Cells were harvested by centrifugation and resuspended in Lysis buffer (20 mM Tris–HCl pH 7.5, 300 mM NaCl and 5 mM β-mercaptoethanol). Cells were lysed using sonification, followed by centrifugation (15 000 g) for 30 min at 8°C. The supernatant was applied to Ni-NTA affinity chromatography for initial purification, followed by size–exclusion chromatography (SEC) purification using gel-filtration columns (HiLoad 16/600 Superdex 200 or 75, GE Healthcare). SDS–PAGE analysis of the proteins was applied in each purification step. Protein concentrations were determined by NanoDrop2000 (ThermoFisher Scientific).

*At*KEOPS complex was reconstituted using purified KAE1–PCC1 and BUD32–CGI121, which were mixed at a molar ratio of 1:2 and applied to SEC for isolation of complete KEOPS complex. *At*KEOPS variants were prepared in same way using corresponding variants of KAE1–PCC1 or BUD32–CGI121. The oligomeric state of *At*KEOPS complex, subcomplexes and variants were determined by SEC (Superdex 200 Increase 10/300 GL column, HiLoad 16/600 Superdex 200 column, GE Healthcare) with reference to *Sc*KEOPS, of which the oligomeric state and molecular weight were determined by SAXS (56). Standard calibration and reference to other well-determined proteins were used for comparing elution volumes and estimating the molecular weight.

### Bulk tRNA extraction

*Arabidopsis thaliana* RNAs were extracted with TRIzol (ThermoFisher Scientific) from seedlings grown for 3 days at 25°C on Murashige and Skoog medium supplemented with 0.8% agar and 1% sucrose, followed by precipitation using absolute ethanol at –80°C. Total RNAs were applied to 8 M urea–polyacrylamide gel (12%) electrophoresis (Urea–PAGE). Gel slices containing full-length tRNAs were cut out, followed by elution in buffer containing 500 mM NaAc pH 5.2 and precipitation in ethanol at –80°C. The tRNA pellets were dissolved in buffer containing 50 mM Tris–HCl pH 8.0, 5 mM MgCl_2_ and 100 mM KCl. Refolding of tRNAs was performed by heating up to 95°C and gradient annealing at a rate of –1°C/min. Yeast total RNAs were extracted with TRIzol from *S. cerevisiae sua5*Δ strain cells that were grown in YPD medium at 28°C for 72 h (4). Bulk *Sc*tRNAs were purified following same protocols of separation, precipitation and refolding as for bulk *At*tRNAs. Concentration of tRNAs was determined by NanoDrop2000 (ThermoFisher Scientific).

### *In vitro* transcription of tRNA

DNA templates of tRNA^Arg^_CCU_ (Gene ID: 3771562), tRNA^Arg^_UCU_ (Gene ID: 3767807), tRNA^Thr^_CGU_ (Gene ID: 3768453), tRNA^Ile^_AAU_ (Gene ID: 3770537), tRNA^Lys^_UUU_ (Gene ID: 3768774), tRNA^Ser^_GCU_ (Gene ID: 3769744), tRNA^Met^_CAU_ (Gene ID: 3766659), tRNA^Asn^_GUU_ (Gene ID: 3768376) and tRNA^Ile^_UAU_ (Gene ID: 3768844) from *Arabidopsis thaliana* were prepared by overlap extension PCR using chemically synthesized primers, in which the forward primer contains a T7 promoter sequence at the 5′ terminus. tRNA genes and primers are summarized in (Supplementary Table S2). Run–off transcription was carried out using T7 RNA polymerase at 30°C for 8 hours in a reaction mixture containing 40 mM Tris–HCl pH 8.0, 5 mM NTP mix, 5 mM DTT, 1 mM spermidine, 0.5% Triton X-100, 33 mM MgCl_2_, 3 μM T7 RNA polymerase, 10 μM pyrophosphatase and 15 mM GMP. RNA transcripts from *in vitro* transcription (IVT) were further purified by Urea–PAGE following protocols as for bulk tRNAs of *Arabidopsis thaliana*. The correct folding of IVT *At*tRNA was confirmed by Circular Dichroism spectra analysis (59). Concentrations of IVT tRNAs were determined by NanoDrop2000.

### Enzymatic synthesis of tRNA t^6^A and LC–MS analysis of t^6^A nucleoside

For TC-AMP formation, 2 μM *At*YRDC was incubated with 4 mM *L*-threonine, 20 mM NaHCO_3_, 2 mM ATP and 5 mM MgCl_2_ in a reaction buffer containing 20 mM Tris–HCl pH 7.5, 200 mM NaCl and 1 mM TCEP for 10 minutes at 25°C. For tRNA t^6^A formation, 2 μM *At*KEOPS and 20 μM tRNA were added to the TC-AMP biosynthesis reaction system and the mixture was incubated for 90 minutes at 30°C followed by purification using 12% Urea–PAGE as described above. The dissolved t^6^A-tRNA was digested into single nucleoside using Nuclease P1 (0.1 U/ml, Sigma) and Alkaline Phosphatase (0.1 U/ml, Sigma). 50 μl sample of the mononucleosides was chromatographed using a C18 column (5 μm, 4.6 × 250 mm, Agilent) at a flow rate of 0.8 ml/min using a mobile phase composed of 0.1% trifluoroacetic acid aqueous and methanol. The nucleosides were isocratically eluted with 5% methanol for 5 min and gradiently eluted with 5–40% methanol for 15 min and 40–98% methanol for 5 min. Nucleosides were identified by the retention time at 254 nm and the mass spectrometry detection (Agilent 6125B). Data collection and analysis were performed using the OpenLab software v3.5 (Agilent). Based on the integrated peak areas of A, U, C, G and t^6^A, the t^6^A modification efficiency was obtained by dividing the peak area ratio (t^6^A/A) by the number (*N*) ratio of 1/(*N*_A_– 1). Three independent replicates were performed for all assays and data graphs were generated using GraphPad Prism. Error bars in quantification data represent standard deviations for triplicate measurements.

### Cryo-EM sample preparation, data collection and processing, model building and validation

800 μg/ml *At*KEOPS was mixed with equal volume of tRNA^Arg^_CCU_ at a molar ratio of 1:2. A drop of 3 μl sample was applied to a freshly glow-discharged Quantifoil gold grid (300 mesh, R0.6/1) and blotted for 2 s at 10°C under 100% humidity in a FEI Vitrobot Mark IV (ThermoFisher Scientific). The grids were stored in the liquid nitrogen until data acquisition. Cryo-EM data were collected on the Titan Krios G3i microscope operated at 300 kV (ThermoFisher Scientific) and equipped with a BioQuantum K3 Imaging Filter direct electron detector (Gatan). The energy filter was used in zero-loss mode with a slit width of 20 eV. All the movies were automatically recorded with EPU software (ThermoFisher Scientific) at a nominal magnification of 105 000 x in super-resolution mode with a pixel size of 0.43 Å. The defocus range was from –0.9 to –2.7 μm. All the movie stacks were collected from three cryo-EM sessions. The total electron dose for each movie stack was 52–64 e^−^/Å^2^ fractionated into 40 fractions over 3.2 s.

The entire image processing was carried out with cryoSPARC v3.3 (60). All the movie stacks were subjected to patch motion correction and patch CTF estimation. A total of 14 622 micrographs were selected to do the following procedure. Firstly, 1 470 particles were manually picked from 100 micrographs with varied defocus values and subjected to 2D classification. Good particles corresponding to those 2D class average maps showing clear density distribution were used for topaz training. In total, 736 244 picks were extracted with a box size of 512 pixels. After several rounds of 2D classification, 327 681 picks were retained and subjected to *ab initio* reconstruction into three models. Heterogeneous refinement was carried out to further clean the data set. A total of 232 232 picks from two classes were combined to perform the homogeneous refinement, yielding a resolution of 3.95 Å. After that, a further round of non-uniform refinement was performed to improve the resolution to 3.65 Å. An overall mask was generated and used in the local refinement, which finally improved the resolution to 3.2 Å. The resolution for all reconstructions were evaluated by using the gold standard Fourier Shell Correlation (FSC) of 0.143. Data collection and reconstruction parameters are presented in Supplementary Table S3.

Prediction structures of *At*KAE1 (AF-O49653-F1), *At*BUD32 (AF-Q94K14-F1), *At*CGI121 (AF-Q6NMZ4-F1) and *At*PCC1 (AF-Q8GWD7-F1) were retrieved from AlphaFold Protein Structure Database (www.alphafold.ebi.ac.uk) (61). *At*KEOPS subcomplexes were generated using crystal structures archaean KEOPS subcomplexes (3,44) and were rigidly fitted into the cryo-EM map using Phenix (62). The complete model was improved with real-space refinement using Phenix, followed by manual building with COOT (63). The final model was validated by MolProbity (64), and the refinement statistics are presented in Supplementary Table S3.

### ATPase assay

The NADH-coupled ATPase assay was employed to analyze the hydrolysis of ATP to ADP (39,47). 200 μl reaction mixture was made of 4 mM phosphoenolpyruvate, 0.5 mM NADH, 6 U/ml pyruvate kinase, 9 U/ml lactate dehydrogenase, 2 mM ATP and 5 μM proteins in the presence or absence of 10 μM tRNA^Arg^_CCU_ in buffer containing 50 mM Tris**–**HCl pH 7.5, 100 mM NaCl, 50 mM KCl, 5 mM MgCl_2_ and 1 mM DTT. The absorbance at 340 nm was recorded at an interval of 30 seconds for a total of 60 min at 25°C with MULTISKAN GO microplate reader (ThermoFisher Scientific) using a 96-well plate. When ATP is hydrolyzed to ADP, NADH is gradually consumed, as reflected by the decrease in absorbance values. A standard curve of ADP against NADH absorbance was obtained to quantify the hydrolysis rate of ATP to ADP. For kinetic analysis, 2 μM protein and 0–50 μM ATP were used and kinetic parameters were calculated according to Michaelis–Menten equation. All the measurements were performed independently in triplicates. Error bars in the data represent standard deviations.

### Electrophoretic mobility shift assay

For the native gel analysis, 20 μM *At*KEOPS was incubated with 20 μM IVT *At*tRNAs in the buffer containing 20 mM Tris–HCl pH 7.5, 300 mM NaCl and 5 mM β-mercaptoethanol and 20% (v/v) glycerol. The mixture was loaded onto a non-denaturating gel (2% agarose, 50 mM Tris–base pH 8.0 and 100 mM glycine) and electrophoresis ran for 1 hour at 100 V at 4°C in pre-chilled buffer containing 50 mM Tris**–**base pH 8.0 and 100 mM glycine. tRNAs were visualized under UV light at 254 nm after staining with Ethidium Bromide, and proteins were stained with Comassie Brilliant Blue. 5′ 6-FAM (6-Carboxyfluorescein)-labelled *At*tRNA^Arg^_CCU_ (5′-6FAM-tRNA^Arg^_CCU_) was chemically synthesized (Tsingke) and was re-folded before use. 0.1 μM 5′-6FAM-tRNA^Arg^_CCU_ was incubated with *At*KEOPS proteins (0–0.5 μM) for gel-shift assay on a non-denaturing gel (1.6% agarose, 50 mM Tris–base pH 8.5 and 100 mM glycine). The electrophoresis ran for 1 hour at 100 V at 4°C in pre-chilled buffer containing 50 mM Tris**–**base pH 8.5 and 100 mM glycine. The presence of 5′-6FAM-tRNA^Arg^_CCU_ on gel was visualized at a wavelength of 488 nm by Pharos FX (Bio-Rad). 10-fold amount of the original input protein in each lane was analyzed and visualized on a separate SDS-PAGE.

### Microscale thermophoresis (MST)

50 nM 5′-6FAM-*At*tRNA^Arg^_CCU_ was incubated with *At*KEOPS complex, subcomplexes or variants at increasing concentrations (0.09765625, 0.1953125, 0.390625, 0.78125, 1.5625, 3.125, 6.25, 12.5, 25, 50 and 100 μM) in MST buffer (20 mM PBS pH 7.5, 300 mM NaCl and 0.05% (v/v) Tween 20). Measurements were performed at 25°C in capillaries (MO-K022, NanoTemper Technologies) on the Monolith NT.115 (NanoTemper Technologies) using 20% LED and medium IR-laser power. Binding data was analyzed using MO.affinity analysis software (NanoTemper Technologies) and equilibrium dissociation constant (*K*_d_) values were fitted by the *K*_d_ equation and model. All experiments were reproduced at least for three times using proteins from different batches. Error bars in data analysis represent standard deviations for triplicate measurements.

### LC–MS/MS proteomic analysis

In the pull-down experiment, 0.5 g *Arabidopsis thaliana* seedlings were grounded in liquid nitrogen. Total proteins were recovered in 200 μl Lysis buffer supplemented with 1 mM PMSF, followed by centrifugation at 4°C. The supernatant was incubated with 100 μg purified *At*KEOPS complex for 1 h. The mixture was applied to purification by Ni-NTA affinity chromatography and unbound proteins were eluted with lysis buffer supplemented with increasing concentrations of imidazole. The final eluted fractions containing KEOPS and potential interactors were denatured and reduced with 2 M guanidine hydrochloride and 10 mM TCEP at 56°C for 30 min. The samples were subsequently alkylated using 40 mM iodoacetamide at room temperature in the dark, followed by overnight digestion using 2 μg trypsin. Tryptic peptides were acidified with 10% formic acid and then loaded on C-18 (3M) stage tips, desalted with 0.1% formic acid and eluted with buffer (80% acetonitrile, 0.1% formic acid). Samples were dried in a vacuum centrifuge. Dried peptides were dissolved in 0.1% formic acid and chromatographed using a C18 column (75 μm inner diameter, 15 cm length, 2 μm particles) on the EASY-nLC1000 UHPLC (ThermoFisher Scientific) coupled to Orbitrap Exploris 480 mass spectrometer (ThermoFisher Scientific). Mass spectra were acquired in data-dependent mode and MS measurements were constructed in the positive-ion mode. The *m/z* range was set to 350–1550, orbitrap resolution is 60 000. MS raw files were processed with the MaxQuant software (version 1.6.14) using standard settings with the additional options match between runs, iBAQ (intensity-based absolute quantification) selected. Other parameters were set up using the default values and the false discovery rate was set to 0.01 for both peptide and protein identification. Relative abundance of the identified interactors was measured by iBAQ values.

### Bioinformatic analysis

We performed the sequence alignment and calculated the sequence identities using Clustal Omega (65). *At*KAE1 (Uniprot: O49653) was aligned to *Homo sapiens* (*Hs*) OSGEP (Uniprot: Q9NPF4), *Saccharomyces cerevisiae* (*Sc*) Kae1 (Uniprot: P36132), *Methanococcus jannaschii* (*Mj*) Kae1 (Uniprot: Q58530), *Pyrococcus abyssi* (*Pa*) Kae1 (Uniprot: Q9UXT7) and *Thermoplasma acidophilum* (*Ta*) Kae1 (Uniprot: Q9HLA5); *At*BUD32 (Uniprot: Q94K14) was aligned to *Hs*PRPK (Uniprot: Q96S44), *Sc*Bud32 (Uniprot: P53323) and *Mj*Bud32 (Uniprot: Q58530); *At*CGI121 (Uniprot: Q6NMZ4) was aligned to *Hs*TPRKB (Uniprot: Q9Y3C4), *Sc*Cgi121 (Uniprot: Q03705) and *Mj*Cgi121 (Uniprot: Q57646); *At*PCC1 (Uniprot: Q8GWD7) was aligned to *Hs*LAGE3 (Uniprot: Q14657), *Sc*Pcc1 (Uniprot: Q3E833), *Pa*Pcc1 (Uniprot: Q9V1Z9) and *Pyrococcus furiosus* (*Pf*) Pcc1 (Uniprot: Q8TZI1). Superposition of structures was performed with COOT and PyMOL (Schrödinger, LLC). Root-mean-square deviation (RMSD) value between two structures was calculated by PyMOL. The visualization and graphic representation of the structures were generated using PyMOL and UCSF Chimera (66). The electrostatic potential molecular surface was generated with Adaptive Poisson–Boltzmann Solver (67).

## Results

### Enzymatic reconstitution of tRNA t^6^A by YRDC and KEOPS from *Arabidopsis thaliana*

We first confirmed t^6^A and a number of other modifications in bulk tRNAs that were isolated from *Arabidopsis thaliana* seedlings (Supplementary Figure S1A). Comparative genomic analysis identified *A. thaliana* (*At*) orthologs of TsaC/Sua5/YRDC―AT5G60590 (YRDC), and four core subunits of KEOPS―AT4G22720 (KAE1), AT5G26110 (BUD32), AT4G34412 (CGI121) and AT5G53045 (PCC1) (11). No orthologs of GON7/Gon7 or Pcc2 were identified to be encoded in *A. thaliana* genome (11,46).

We first constructed an expression plasmid for *A. thaliana* YRDC using chemically synthesized DNA and purified recombinant YRDC (Supplementary Figure S1B) that was expressed bacterial cells. Liquid chromatography–mass spectrometry (LC–MS) analysis shows that YRDC is active in catalyzing the formation of threonylcarbamoyl-AMP (TC-AMP) in the presence of ATP, *L*-threonine and NaHCO_3_ (Supplementary Figure S1B).

**Figure 1.**
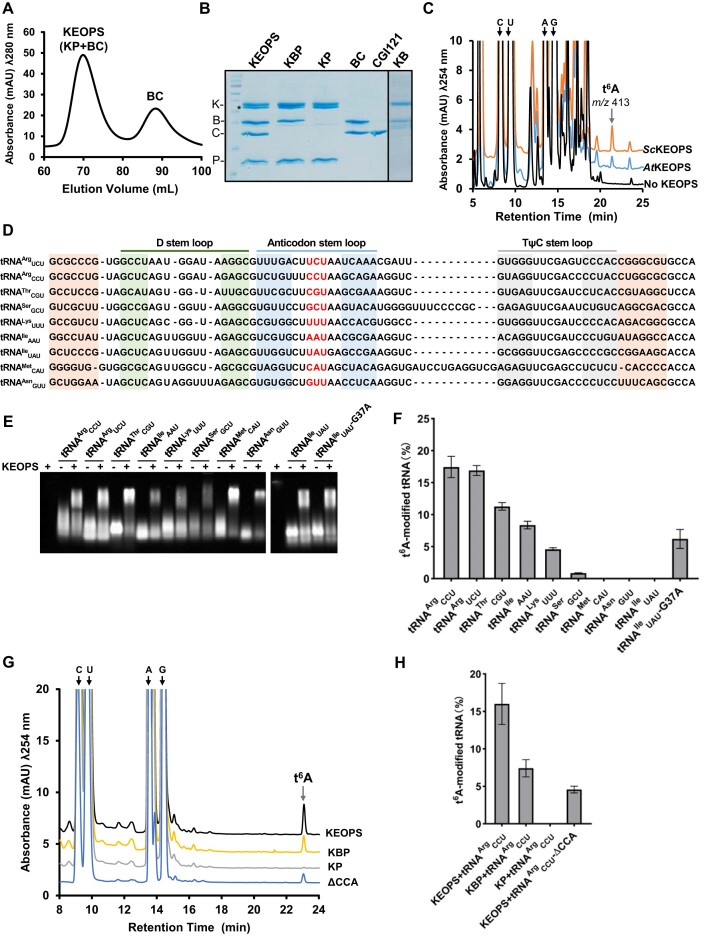
*In vitro* enzymatic reconstitution of tRNA t^6^A biosynthesis using recombinant YRDC and KEOPS proteins of *Arabidopsis thaliana*. (**A**) Size-exclusion chromatography (SEC) profile (HiLoad 16/600 Superdex 200, GE Healthcare) of *A. thaliana* KEOPS and BUD32–CGI121. KEOPS was reconstituted using purified KP (KAE1–PCC1) and BC (BUD32–CGI121), which were mixed at a molar ratio of 1:2. (**B**) SDS–PAGE analysis of KEOPS, KBP (KAE1–BUD32–PCC1), KP, BC, CGI121 and KB (KAE1–BUD32). * indicates KAE1 degradation (confirmed by LC–MS/MS). (**C**) LC–MS analysis of t^6^A formation in assay that contained 4 mM l-threonine, 20 mM NaHCO_3_, 2 mM ATP, 20 μM bulk *Sc*tRNAs (isolated from *sua5*Δ strain), 2 μM *At*YRDC and 2 μM *At*KEOPS or *Sc*KEOPS. (**D**) Nucleotide sequences of *A. thaliana* tRNAs that were *in vitro* transcribed (IVT) and used for tRNA t^6^A assays. Amino acid acceptor arm, D stem loop, anticodon stem loop and TψC stem loop of tRNAs are highlighted in pink, green, blue and gray, respectively. Anticodons are shown in red. (**E**) Native gel analysis of the interaction between 10 μM *At*KEOPS and 20 μM IVT *At*tRNAs (tRNA^Arg^_CCU_, tRNA^Arg^_UCU_, tRNA^Thr^_CGU_, tRNA^Ile^_AAU_, tRNA^Lys^_UUU_, tRNA^Ser^_GCU_, tRNA^Met^_CAU_, tRNA^Asn^_GUU_, tRNA^Ile^_UAU_ and tRNA^Ile^_UAU_-G37A). (**F**) Quantified t^6^A modification efficiencies of *At*KEOPS towards these IVT *At*tRNAs. Percentage of t^6^A was normalized according to tRNA sequence. (**G**) LC–MS analysis of tRNA t^6^A formation by 2 μM *At*YRDC and *At*KEOPS, *At*KBP or *At*KP using 20 μM IVT *At*tRNA^Arg^_CCU_ or *At*tRNA^Arg^_CCU_-ΔCCA as substrate. (**H**) Quantified t^6^A modification efficiencies of *At*KEOPS, *At*KBP and *At*KP towards *At*tRNA^Arg^_CCU_ or *At*tRNA^Arg^_CCU_-ΔCCA (G).

We constructed expression plasmids of *A. thaliana* KEOPS proteins using chemically synthesized DNAs and tried heterologous expression of KAE1–BUD32–PCC1–CGI121 (KEOPS), BUD32–CGI121 (BC), KAE1–PCC1 (KP), KAE1–BUD32 (KB), KAE1–BUD32–PCC1 (KBP) and CGI121 (C). We did not succeed in concomitantly expressing KAE1, BUD32, CGI121 and PCC1 using a polycistronic plasmid similar to that for *S. cerevisiae* (*Sc*) KEOPS (56). Alternatively, we assembled KEOPS by mixing KP and BC at a molar ratio of 1:2, followed by purification using size―exclusion chromatography (SEC) (Figure 1A). The SEC and SDS–PAGE analysis of KEOPS complex, subcomplexes (KBP, KP, BC, KB) and CGI121 are shown in Supplementary Figure S1C and Figure 1B, respectively. Notably, we observed a persistent degradation KAE1 via the N-terminal end, which was confirmed by MS and western blot against 6His tag. SEC analysis shows that the four-subunit *At*KEOPS eluted out (at a calculated molecular weight of ∼200 kDa) earlier than the five-subunit *Sc*KEOPS (a monomeric complex with a molecular weight of 117.7 kDa) (56), indicating that *At*KEOPS exists as an eight-subunit dimer (K_2_B_2_C_2_P_2_) as that of *Mj*KEOPS (44) (Supplementary Figure S1C). Compared SEC profiles suggest that either KAE1–PCC1 or KAE1–BUD32–PCC1 forms a dimer whereas BUD32–CGI121 or KAE1–BUD32 exists as a monomer (Supplementary Figure S1C).

We first tested the t^6^A-catalytic activity of *At*KEOPS using bulk *Sc*tRNAs that were isolated from *S. cerevisiae sua5*Δ cells. LC–MS analysis shows that *Sc*tRNAs were deficient in t^6^A but acquired t^6^A in the presence of *At*KEOPS and *At*YRDC (Figure 1C). In addition, the catalytic activity of *At*KEOPS towards *Sc*tRNAs was comparable to that of *Sc*KEOPS (Figure 1C). In summary, the active recombinant proteins expressed in *E. coli* cells demonstrate that folding and function of KEOPS do not require essential post-translational modifications for *in vitro* biosynthesis of tRNA t^6^A. We transcribed and purified 9 ANN-decoding tRNAs―tRNA^Arg^_UCU_, tRNA^Arg^_CCU,_ tRNA^Thr^_CGU_, tRNA^Ser^_GCU_, tRNA^Lys^_UUU_, tRNA^Ile^_AAU_, tRNA^Ile^_UAU_, tRNA^Met^_CAU_ and tRNA^Asn^_GUU_―that are encoded in *A. thaliana* nuclear genome (Figure 1D). Electrophoretic mobility shift assay (EMSA) confirmed an interaction between *At*KEOPS and each of these *in vitro* transcribed (IVT) tRNAs (Figure 1E). We then performed t^6^A assays using these IVT *At*tRNAs and analyzed t^6^A formation by LC–MS analysis (Supplementary Figure S1D). Normalized levels of t^6^A against A, U, C or G according to the nucleotide sequence showed great variations in t^6^A modification efficiencies of *At*KEOPS towards these IVT *At*tRNAs (Figure 1F). *At*KEOPS exhibits strong activities on tRNA^Arg^_CCU_ and tRNA^Arg^_UCU_, and gradually lowering activities on tRNA^Thr^_CGU_, tRNA^Ile^_AAU_, tRNA^Lys^_UUU_ and tRNA^Ser^_GCU_. In comparison, tRNA^Met^_CAU_, tRNA^Asn^_GUU_ and tRNA^Ile^_UAU_ were not t^6^A-modified by *At*KEOPS. To exclude the possible problem in preparation of IVT tRNAs, we also deterimined different t^6^A-modification variations in these IVT *At*tRNAs by *Sc*KEOPS (Supplementary Figure S1E and F). Our analysis suggests that the t^6^A modification frequencies are dependent on sequences of *At*tRNAs. In our case, *At*tRNA^Ile^_UAU_ and *At*tRNA^Met^_CAU_ lack a 36-UAA-38 motif (Figure 1D), which is a determinant of t^6^A modification (55). We mutated 36-UGA-38 motif of *At*tRNA^Ile^_UAU_ (Figure 1F) into a 36-UAA-38 motif and generated a tRNA^Ile^_UAU_ variant―*At*tRNA^Ile^_UAU_-G37A. Indeed, *At*tRNA^Ile^_UAU_-G37A is t^6^A-modified by *At*KEOPS (Figure 1F). As for the inactivity of *At*tRNA^Asn^_GUU_, we confirmed the overall folding by CD spectra analysis (Supplementary Figure S1D). Promoted by a recent discovery of a novel function of *P. salinus* TsaN in generating t^6^ATP/t^6^ADP using TC-AMP and ATP/ADP (47), we also analyzed the formation of t^6^ATP/t^6^ADP by KEOPS via detecting t^6^A―the dephosphorylated products of t^6^ATP/t^6^ADP. Our LC–MS analysis shows that *At*KEOPS is inactive in generating t^6^ATP/t^6^ADP in the presence of ATP and TC-AMP while *P. salinus* TsaN^1–392^ (TsaD domain) catalyzes the formation of t^6^ATP/t^6^ADP in conjunction with *At*YRDC (Supplementary Figure S1B).

As *At*KEOPS possesses the highest activity on *At*tRNA^Arg^_CCU_, we set out to test the t^6^A-catalytic activities of *At*KEOPS subcomplexes in assays using IVT *At*tRNA^Arg^_CCU_ as substrate (Figure 1G and 1H). Our data demonstrates that KAE1–BUD32–PCC1 subcomplex is still capable of catalyzing the t^6^A formation of *At*tRNA^Arg^_CCU_, but the activity is decreased by around 50% compared that of KEOPS. KAE1–PCC1 subcomplex is no longer active in catalyzing the t^6^A modification of *At*tRNA^Arg^_CCU_ (Figure 1F). In addition, we show that *At*KEOPS still catalyzes t^6^A-modification of tRNA^Arg^_CCU_ΔCCA in which the 3′ CCA end was chopped (Figure 1G and 1H). In sum, our data show that BUD32 is strictly needed by *At*KEOPS to catalyze the biosynthesis of tRNA t^6^A while CGI121 is dispensable for *in vitro* tRNA t^6^A biosynthesis. In contrast, *Mj*Cgi121 directly binds tRNA via the 3′ CCA end and plays a pivotal role in tRNA binding and t^6^A-catalytic function of *Mj*KEOPS (45).

### Cryo-EM structure of *A. thaliana* KEOPS complex

**Figure 2.**
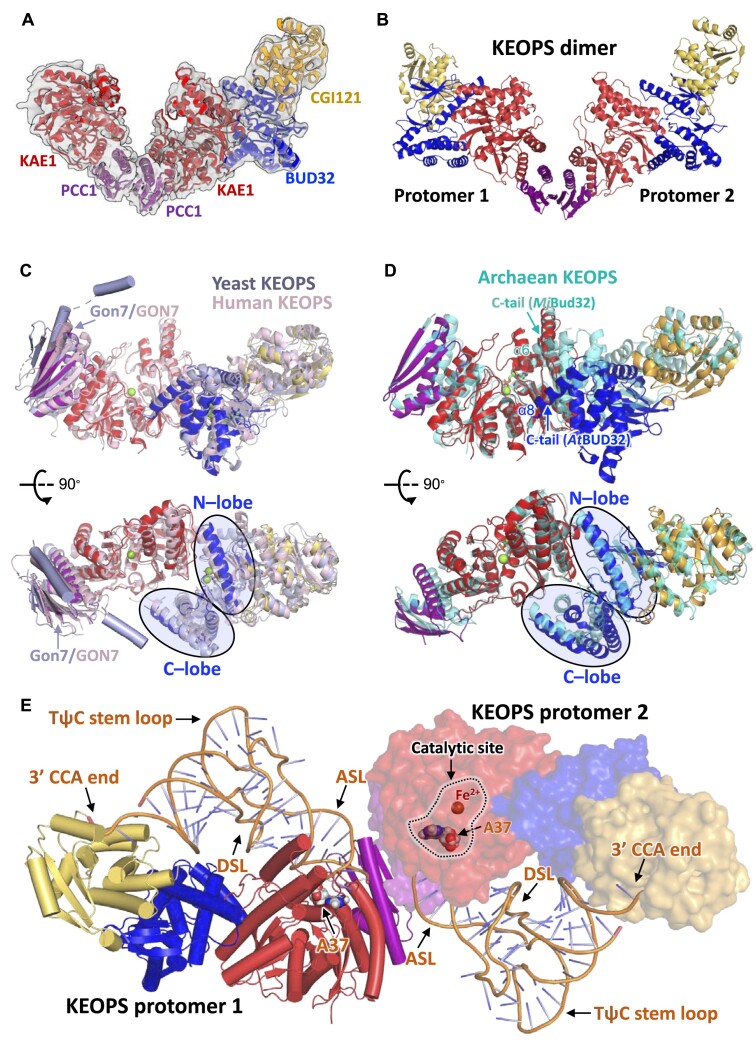
Cryo-EM structure of *A. thaliana* KEOPS complex. (**A**) Cartoon representation of the cryo-EM structure of *A. thaliana* KEOPS complex (KAE1–PCC1–PCC1–KAE1–BUD32–CGI121) associated with cryo-EM map that is represented in transparent surface. PCC1, KAE1, BUD32 and CGI121 are color-coded in purple, red, blue and orange, respectively. (**B**) Reconstructed model of *A. thaliana* KEOPS dimer. BUD32–CGI121 from protomer 1 was modelled to complete protomer 2 via structural alignment of PCC1–KAE1. (**C**) Structural comparison of *S. cerevisiae* KEOPS (Gon7–Pcc1–Kae1–Bud32–Cgi121), *H. sapiens* KEOPS (GON7–LAGE3–OSGEP–PRPK–TPRKB) and *A. thaliana* KEOPS (PCC1–KAE1–BUD32–CGI121). Crystal structures of Bud32–Cgi121 (PDB: 4WW9)/PRPK–TPRKB (PDB: 6WQX) and Gon7–Pcc1 (PDB: 4WX8)/GON7–LAGE3–OSGEP (PDB: 6GWJ) were aligned to structures of their orthologs in *A. thaliana* KEOPS complex. Structures of Gon7/GON7 at the extremity are represented in cylindrical helices. (**D**) Structural alignment of KAE1 in *A. thaliana* KEOPS complex (PCC1–KAE1–BUD32–CGI121) and Kae1 in archaean KEOPS complex (Pcc1–Kae1–Bud32–Cgi121), of which a composite model was generated using crystal structure of *M. jannaschii* Kae1–Bud32–Cgi121 (PDB: 3ENH) and crystal structure of *M. jannaschii* Kae1–*P. furiosus* Pcc1 (PDB: 5JMV). Two different conformations of the C-lobes and C-terminal tails of *A. thaliana* BUD32 and *M. jannaschii* Bud32 are indicated with arrows. (**E**) A model of *A. thaliana* KEOPS–tRNA complex was generated by structural juxtaposition of *A. thaliana* KEOPS dimer and archaean KEOPS–tRNA, of which the model was generated using a composite model of archaean KEOPS complex and a crystal structure of *M. jannaschii* Cgi121–tRNA^Lys^_UUU_ (PDB: 7KJT). The model of archaean KEOPS (D) was omitted for clarity. D stem loop (DSL), anticodon stem loop (ASL), TψC stem loop and 3′ CCA end of tRNA are labeled.

We set out to determine the structure of *At*KEOPS–tRNA^Arg^_CCU_ by single particle cryo-electron microscopy (cryo-EM). We were not able to isolate a preformed *At*KEOPS–tRNA^Arg^_CCU_ complex by SEC (data not shown). Alternatively, a mixture of freshly purified *At*KEOPS and *At*tRNA^Arg^_CCU_ (at a molar ratio of 1:2) was applied to cryo-EM grids for structural analysis. We optimized the vitrification and collected a dataset of 14 622 micrographs on a Titan Krios microscope operated at 300 keV. We analyzed the complete dataset by applying manual picking and topaz training as well as iterative rounds of particle picking. With 232 232 picks, we determined a map of *At*KEOPS at an overall resolution of 3.2–5.5 Å (Supplementary Figure S2 and Supplementary Table S3). We retrieved prediction models of *At*KEOPS subunits from AlphaFold Protein Structure Database (61) and generated fitting models for subcomplexes of KAE1–PCC1, KAE1–BUD32, BUD32–CGI121 and PCC1–PCC1 based on crystal structures of *Mj*Kae1–Pcc1 (44), *Mj*Kae1–Bud32 (3), *Sc*Bud32–Cgi121 (56) and *P. furiosus* (*Pf*) Pcc1–Pcc1 (44), respectively. The reconstruction shows six KEOPS subunits―KAE1, PCC1, PCC1, KAE1, BUD32 and CGI121, but no presence of a tRNA molecule (Figure 2A). The dissociation of BUD32–CGI121 from KAE1–PCC1 in one KEOPS protomer was probably due to the structural damage at air–water interface during sample preparation. Imposing local refinement (Supplementary Figure S2B and C), we managed to determine almost a complete structure of *At*KEOPS complex with exception for the C-terminal tail of KAE1 (residues 341–353), N-terminal end (residues 1–13) and C-terminal tail (residues 222–226) of BUD32, and N-terminal end of PCC1 (residues 1–15). The map was blurred for two flexible loops (residues 36–48 and 173–179) of KAE1, which were modelled with references to crystal structures of its orthologs (Supplementary Figure S3A). We modelled a Fe^2+^ ion in the binding sphere of H113–H117–D298 motif in KAE1 based on crystal structures of Kae1/OSGEP proteins (3,18,52). The final model is composed of a complete *At*KEOPS protomer (PCC1–KAE1–BUD32–CGI121) and an incomplete protomer that only contains KAE1–PCC1 (Figure 2A). Based on the dimeric state of *At*KEOPS complex in solution as determined by biochemical characterizations (Figure 1B and Supplementary Figure S1C), we aligned the structure of a complete KEOPS protomer to the structure of KAE1–PCC1 and completed a model of BUD32–CGI121 in relation to KAE1–PCC1 in the other protomer. In the reconstructed model of *At*KEOPS dimer, the two protomers interact via an interacting interface of PCC1 dimer and adopt a V-shaped architecture (Figure 2B).

### The structural uniformity and variation of KEOPSs

KEOPSs function as molecular machineries (31). Up to date, no atomic structures of a complete KEOPS complex are available except for a composite model of KEOPS from archaea (3) and crystal structures of KEOPS subcomplexes from yeast (56) and humans (18,57). We compared these KEOPS structures with *At*KEOPS to gain insights into functions. Overall, *At*KEOPS subunits manifest a substantial structural conservation to their orthologs from archaea, yeast and humans (Supplementary Figure S3). RMSD values of structural alignment coupled sequence identities are summarized in Table 1. Crystal structures of *Hs*LAGE3–OSGEP binary complex (18) and *Hs*PRPK–TPRKB binary complex (57) are perfectly superposed with structure of *At*KEOPS (Figure 2C). Likewise, crystal structures of *Sc*Pcc1 (56) and *Sc*Bud32–Cgi121 binary complex (56) are satisfactorily aligned with structure of *At*KEOPS (Figure 2C) except for Kae1 whose structure has not been determined. As for archaean KEOPS, crystal structures of *Pf*Pcc1–*Mj*Kae1 binary complex and *Mj*Bud32–Cgi121 binary complex are also satisfactorily superposed to PCC1–KAE1 and BUD32–CGI121 in the cryo-EM structure of *At*KEOPS, respectively (Supplementary Figure S3E). In sum, the overall arrangement and inter-subunit interfaces of KEOPS subunits are extremely conserved among KEOPSs. However, structural juxtaposition of *At*KEOPS and *Mj*KEOPS as a whole reveals a marked variation in the C-lobes of BUD32 and Bud32 (Figure 2D). In general, the C-lobe of *At*BUD32 is located farther away (at least 4 Å) than that of *Mj*Bud32 from the C-terminal domain of KAE1. Consequently, C-lobe makes fewer contacts with KAE1 (Figure 2D). Superposition of *At*PCC1 dimer and *Pf*Pcc1 dimer exhibits conserved V-shaped architectures but varied conformations of *At*KEOPS dimer and *Mj*KEOPS dimer (Supplementary Figure S3F).

**Table 1.** Statistics of the structural alignment coupled sequence identities for KEOPS subunits from *A. thaliana*, *H. sapiens*, *S. cerevisiae* and archaean species (*M. jannaschii, P. abyssi, T. acidophilum* and *P. furiosus*)

	Comparing protein (PDB ID)	Sequence identity (%)	RMSD (Å)
**KAE1**	*H. sapiens* OSGEP (6GWJ)	68.95	1.074 (379 C_α_)
	*M. jannaschii* Kae1 (5JMV)	44.19	1.236 (288 C_α_)
	*P. abyssi* Kae1 (2IVP)	46.91	1.248 (292 C_α_)
	*T. acidophilum* Kae1 (3ENO)	41.31	1.515 (297 C_α_)
**BUD32**	*H. sapiens* PRPK (6WQX)	43.80	1.852 (178 C_α_)
	*S. cerevisiae* Bud32 (4WW9)	28.31	1.689 (151 C_α_)
	*M. jannaschii* Bud32 (3ENH)	30.85	1.996 (154 C_α_)
**CGI121**	*H. sapiens* TPRKB (6WQX)	29.65	1.208 (127 C_α_)
	*S. cerevisiae* Cgi121 (4WW9)	16.86	1.471 (116 C_α_)
	*M. jannaschii* Cgi121 (7KJT)	13.10	3.026 (119 C_α_)
**PCC1**	*H. sapiens* LAGE3 (6GWJ)	33.33	1.057 (73 C_α_)
	*S. cerevisiae* Pcc1 (4WXA)	25.00	1.014 (60 C_α_)
	*P. abyssi* Pcc1 (7A67)	15.85	1.469 (47 C_α_)
	*P. furiosus* Pcc1 (5JMV)	14.63	1.611 (63 C_α_)

Unfortunately, tRNA^Arg^_CCU_ was not resolved in the cryo-EM structure of *At*KEOPS. We made use of *Mj*KEOPS–tRNA model to gain insights into the binding of tRNA to *At*KEOPS. Structural juxtaposition of *Mj*KEOPS–tRNA to *At*KEOPS dimer generated a model for a dimer of *At*KEOPS–tRNA (Figure 2E). According to this model, tRNA could be accommodated on an extended surface of the four subunits of *At*KEOPS. According to the docked model, anticodon stem loop of tRNA is sandwiched between KAE1 and PCC1; D stem loop protrudes between KAE1 and BUD32; 3′ CCA end extends to nucleotides-binding pocket of CGI121. Though steric clashes in some regions are evident, e.g. KAE1 and anticodon stem loop, this model might represent a general binding orientation of tRNA to *At*KEOPS based on *Mj*KEOPS–tRNA model (45).

### KEOPS dimer is required to form a t^6^A-catalytic KEOPS–tRNA assembly

**Figure 3.**
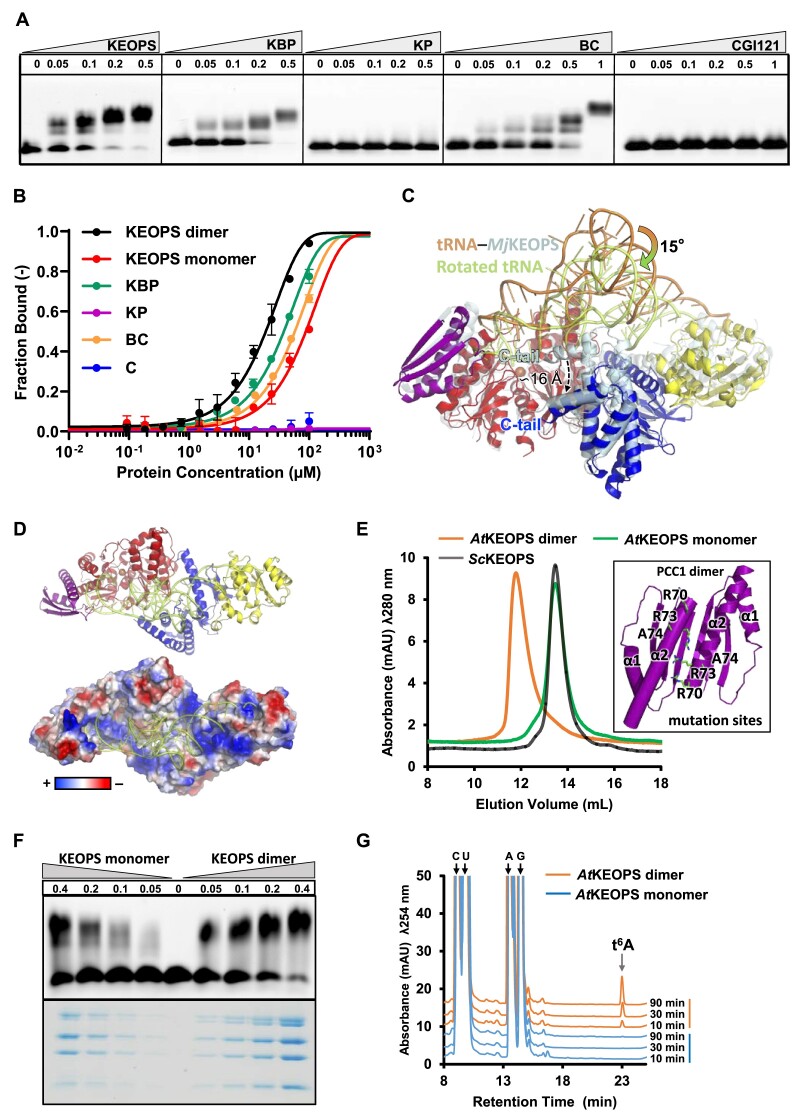
Characterization of the interaction between *A. thaliana* KEOPS and tRNA. (**A**) EMSA analysis of the interaction between 0.1 μM 5′-6FAM-tRNA^Arg^_CCU_ and 0–0.5 μM *A. thaliana* KEOPS complex and subcomplex: KAE1–BUD32–PCC1 (KBP), KAE1–PCC1 (KP), BUD32–CGI121 (BC) and CGI121 (C). The gels show migration of 0.1 μM 5′-6FAM-tRNA^Arg^_CCU_ in the absence of KEOPS proteins at indicated concentrations. (**B**) Microscale thermophoresis (MST) measurements of the interactions between 50 nM 5′-6FAM-tRNA^Arg^_CCU_ and *A. thaliana* KEOPS proteins at indicated concentrations (97 nM–100 μM). KEOPS dimer, wild-type KEOPS; KEOPS monomer, P^R70D/R73D/A74E^KBC. Error bars represent standard deviations for triplicate measurements. (**C**) A rigidly adjusted model of *A. thaliana* KEOPS–tRNA complex in which tRNA (colored in orange) from archaean KEOPS–tRNA was rotated by ∽15° along with structural alignment (shifted by ∽16 Å) of the C-terminal lobe of *M. jannaschii* Bud32 (represented in cylindrical helices) to that of *A. thaliana* BUD32. (**D**) Geometrical complementarity and electrostatic potential surface representation of *A. thaliana* KEOPS–tRNA model. (**E**) SEC (Superdex 200 Increase 10/300 GL, GE Healthcare) profiles of *A. thaliana* KEOPS dimer (P_2_K_2_B_2_C_2_), KEOPS monomer (P^R70D/R73D/A74E^KBC) and *S. cerevisiae* KEOPS. The insert shows the interacting interface of *A. thaliana* PCC1 dimer and sites of mutation. (**F**) EMSA analysis of the interactions between 0.1 μM 5′-6FAM-tRNA^Arg^_CCU_ and 0–0.4 μM *At*KEOPS dimer or KEOPS monomer (P^R70D/R73D/A74E^KBC). The upper panel shows the migration of 5′-6FAM-tRNA^Arg^_CCU_ and the lower panel shows 10-fold amount of the original protein input in each lane as visualized by a separate SDS–PAGE. (**G**) LC–MS analysis of the time-course formation of t^6^A in assay that contained 2 μM *A. thaliana* KEOPS dimer or KEOPS monomer, 5 μM *A. thaliana* YRDC and 20 μM IVT *At*tRNA^Arg^_CCU_. The assay contained 4 mM *L*-threonine, 20 mM NaHCO_3_ and 2 mM ATP.

Based on high t^6^A-catalytic activity of *At*KEOPS on *At*tRNA^Arg^_CCU_, we chose to use 5′ 6-FAM (6-Carboxyfluorescein)-labelled tRNA^Arg^_CCU_ (5′-6FAM-tRNA^Arg^_CCU_) for interaction analysis. Our gel analysis shows a strong interaction between *At*KEOPS and 5′-6FAM-tRNA^Arg^_CCU_ (Figure 3A). In contrast, neither PCC1–KAE1 nor CGI121 is capable of binding 5′-6FAM-tRNA^Arg^_CCU_. In addition, our analysis revealed a relatively weak interaction between 5′-6FAM-tRNA^Arg^_CCU_ and PCC1–KAE1–BUD32 or BUD32–CGI121 (Figure 3A). We further measured the binding affinities with microscale thermophoresis (MST) (Figure 3B) and determined equilibrium constants (*K*_d_) for the interactions between 5′-6FAM-tRNA^Arg^_CCU_ and *At*KEOPS proteins (Table 2). We determined a *K*_d_ value of 18.08 μM for the interaction between 5′-6FAM-tRNA^Arg^_CCU_ and *At*KEOPS. In comparison, *K*_d_ values for PCC1–KAE1–BUD32 and BUD32–CGI121 towards 5′-6FAM-tRNA^Arg^_CCU_ are 37.99 μM and 61.58 μM, respectively. Consistent with EMSA analysis, no binding events were observed with 5′-6FAM-tRNA^Arg^_CCU_ and PCC1–KAE1 or CGI121 by MST measurements (Figure 3B). These binding data demonstrate that CGI121 does not bind tRNA but promotes the binding of tRNA to KEOPS whereas BUD32 is strictly needed for binding of tRNA to *At*KEOPS.

**Table 2.** Summary of the equilibrium dissociation constants (*K*_d_) for the binding of 5′-6FAM-tRNA^Arg^_CCU_ to *A. thaliana* KEOPS proteins as determined by microscale thermophoresis (MST). K, KAE1; B, BUD32; C, CGI121; P, PCC1; dimer, wild-type KEOPS complex (P_2_K_2_B_2_C_2_); monomer, KP^R70D/R73D/A74E^BC; mutant-1, Y36R/I37K/T38R/P39R/P40R/G41W/H42D; mutant-2, G43E/F44R/L45K/P46R/R47D/E48K; n.d., not determined

KEOPS	*K*_d_ (μM)	KAE1 mutants	*K*_d_ (μM)	BUD32 mutants	*K*_d_ (μM)
**dimer**	18.08 ± 3.87	**K^H117A^BCP**	53.75 ± 17.70	**KB^I47K^CP**	221.59 ± 216.78
**monomer**	74.37 ± 13.51	**K^D298R^BCP**	97.90 ± 39.83	**KB^K51E^CP**	29.82 ± 10.66
**KBP**	37.99 ± 13.56	**K^I17F^BCP**	315.36 ± 16.72	**KB^K55E^CP**	104.29 ± 15.22
**KP**	n.d.	**K^K202R^BCP**	111.31 ± 21.71	**KB^N58R^CP**	9.81 ± 2.73
**BC**	61.58 ± 15.02	**K^R284C^BCP**	128.18 ± 5.68	**KB^T162R^CP**	10.47 ± 2.99
**CGI121**	n.d.	**K^A231G^BCP**	138.89 ± 51.80	**KB^S163R^CP**	329.68 ± 11.10
**KBCP^R73D^**	352.7 ± 39.96	**K^Y305A^BCP**	71.18 ± 13.80	**KB^L165K^CP**	302.42 ± 200.43
–	–	**K^mutant-1^BCP**	5.27 ± 1.20	**KB^R220stop^CP**	148.39 ± 26.97
–	–	**K^mutant-2^BCP**	n.d.	**KB^R220A^CP**	88.20 ± 15.58

Our interaction analysis demonstrates a central role of BUD32 in tRNA binding. However, the model of *At*KEOPS–tRNA does not support a direct contact between BUD32 and tRNA (Figures 2E and 3C). In contrast, the model of *Mj*KEOPS–tRNA coupled mutational validations demonstrated that the C-lobe of *Mj*Bud32 interacts with D stem loop and amino acid acceptor stem of *Mj*tRNA^Lys^_UUU_ (45). We presumed that the C-lobe of *At*BUD32 is also primarily involved in binding of tRNA. We manually adjusted the position of tRNA by means of aligning the C-lobe of *Mj*Bud32 in rigid complex with *Mj*tRNA^Lys^_UUU_ to the C-lobe of *At*BUD32, leading to an adjusted model of *At*KEOPS–tRNA (Figure 3C). In this model, tRNA was rotated by ∽15° and shifted by ∽16 Å towards the C-lobe of *At*BUD32, giving better geometrical complementarity and electrostatic interactions (Figure 3D). Such a manual rotation of *Mj*tRNA^Lys^_UUU_ from crystal structure of *Mj*Cgi121–tRNA^Lys^_UUU_ was also performed to fit the geometry of *Mj*KEOPS (45). It further suggests that assembly of KEOPS–tRNA complex might involve large conformational changes in KEOPS subunits and tRNA.

We set out to find out whether *At*KEOPS dimer is needed for tRNA t^6^A biosynthesis. To disrupt the PCC1 dimer, we simultaneously mutated the Arg70, Arg73 and Ala74 of PCC1 (Figure 3E) and generated an *At*KEOPS variant―PCC1^R70D/R73D/A74E^–KAE1–BUD32–CGI121 (P^R70D/R73D/A74E^KBC) (Supplementary Figure S4A and B). SEC analysis demonstrates that P^R70D/R73D/A74E^KBC exists exclusively as a monomer with an elution volume roughly overlapping with that of the five-subunit *Sc*KEOPS (Figure 3E). Hereafter, P^R70D/R73D/A74E^KBC is dubbed as *At*KEOPS monomer and the dimer refers to wild-type *At*KEOPS. The interaction between *At*KEOPS monomer and 5′-6FAM-tRNA^Arg^_CCU_ becomes apparently weaker than that of *At*KEOPS dimer and 5′-6FAM-tRNA^Arg^_CCU_ (Figure 3F). MST measurements determined a *K*_d_ value of 74.37 μM for the interaction between *At*KEOPS monomer and 5′-6FAM-tRNA^Arg^_CCU_ (Figure 3B). Our LC–MS analysis shows that *At*KEOPS monomer is no longer active in catalyzing t^6^A biosynthesis (Figure 3G). To find out whether the loss of t^6^A-ctalytic activity is due to disruption of dimer but not the loss of Arg70, Arg73 or Ala74 (as functional sites), we mutated Arg73 of PCC1 and generated an *At*KEOPS variant―PCC1^R73D^–KAE1–BUD32–CGI121 (P^R73D^KBC). SEC analysis shows that P^R73D^KBC elutes out between *At*KEOPS dimer and monomer (Supplementary Figure S4A and B), suggesting that P^R73D^KBC exists as mixture of dimer and monomer. Another explanation of the SEC profile is that the R73D mutation possibly induces large conformational changes in the dimeric architecture. EMSA analysis and MST measurements exhibit a significantly weaker interaction (*K*_d_= 352.7 μM) between P^R73D^KBC and 5′-6FAM-tRNA^Arg^_CCU_ (Supplementary Figure S4C and D). However, P^R73D^KBC still sustains around 25% t^6^A-catalytic activity of *At*KEOPS dimer (Supplementary Figure S4E). In sum, our structural analysis and functional validation show that *At*KEOPS dimer is needed to form a t^6^A-catalytic KEOPS–tRNA assembly.

### Characterization KAE1 reveals regulatory sites related to t^6^A-catalytic activity of KEOPS

**Figure 4.**
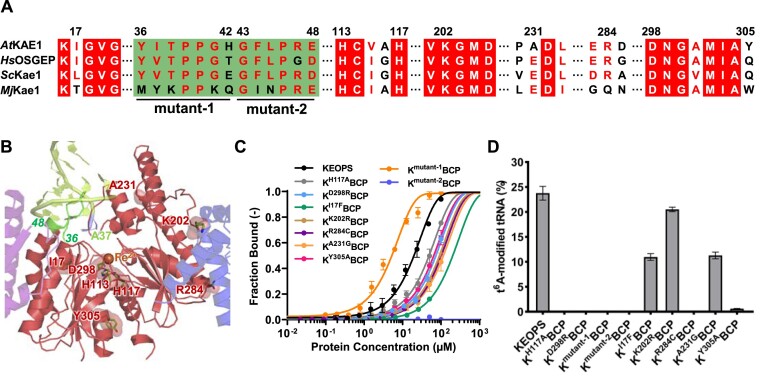
Characterization of functional sites of KAE1. (**A**) Local sequence alignment of *A. thaliana* (*At*) KAE1, *H. sapiens* (*Hs*) OSGEP, *S. cerevisiae* (*Sc*) Kae1 and *M. jannaschii* (*Mj*) Kae1. (**B**) The sites of single-mutation (I17, H117, K202, A231, R284, D298 and Y305) and the connecting loop (residues 36–48) of α1 and β1 are labeled in the structure of KAE1 (colored in red). (**C**) Microscale thermophoresis (MST) measurements of the interactions between 50 nM 5′-6FAM-tRNA^Arg^_CCU_ and *At*KEOPS variants bearing mutations in KAE1. mutant-1, Y36R/I37K/T38R/P39R/P40R/G41W/H42D; mutant-2, G43E/F44R/L45K/P46R/R47D/E48K. Error bars represent standard deviations for triplicate measurements. (**D**) Comparison of t^6^A modification efficiencies of IVT *At*tRNA^Arg^_CCU_ by *At*KEOPS variants bearing mutations in KAE1 in conjunction with 2 μM *At*YRDC.

KAE1 adopts a typical two-subdomain fold that is conserved in all TsaD/Kae1/Qri7/OSGEP structures (Supplementary Figure S3A). The t^6^A-catalytic site is located between the two subdomains and a divalent metal ion (Fe^2+^/Zn^2+^) is essential for TC-AMP binding and t^6^A catalysis (32,41,54). Based on the model of KEOPS–tRNA complex (Figure 3C) and loss-of-function mutations in *Hs*OSGEP, we chose to characterize the functional sites of *At*KAE1 (Figure 4B): (i) an extremely conserved H–H–D motif (H117 and D298) that coordinates the metal ion Fe^2+^ (32); (ii) I17, K202 and R284 that are equivalent to missense mutations (I14F, K198R, R280C) of *Hs*OSGEP in GAMOS patients (16); (iii) non-conserved A231 and Y305 in the vicinity of the catalytic site of KAE1. We generated these mutations and purified corresponding KEOPS variants: K^H117A^BCP, K^D298R^BCP, K^I17F^BCP, K^K202R^BCP, K^R284C^BCP, K^A231G^BCP and K^Y305A^BCP (Supplementary Figure S5A and B). Our gel interaction analysis shows that 5′-6FAM-tRNA^Arg^_CCU_ is almost completely bound to these KEOPS variants when mixed at a molar ratio of 1:5 (Supplementary Figure S5C). However, quantitative analysis by MST reveals that the interactions between 5′-6FAM-tRNA^Arg^_CCU_ and these KEOPS variants are weaker than that between 5′-6FAM-tRNA^Arg^_CCU_ and wild-type KEOPS (Figure 4C), as indicated by increased *K*_d_ values for these KEOPS variants (Table 2). Notably, I17F severely interferes with the binding of tRNA to KEOPS. We performed assays on t^6^A catalysis of these KEOPS variants (Supplementary Figure S5E and F) and show different effects of these mutations on t^6^A-catalytic activity of KEOPS (Figure 4D): 1) K202R mutation confers less effect on t^6^A-catalytic activity of KEOPS; 2) either I17F mutation or A231G mutation results in half of the t^6^A-catalytic activity; 4) H117A, D298R or R284C leads to dead t^6^A-catalytic activity; 5) Y305A mutation also causes a dead t^6^A-catalytic activity as only trace amount of t^6^A was detected. In light of the structural model (Figure 4B), our analysis of the interaction and t^6^A-catalysis of these mutations of KAE1 confirms an essential role of H117 and D298 in t^6^A catalysis. Interestingly, no degradation of KAE1 was observed in K^H117A^BCP and K^D298R^BCP (Supplementary Figure S5B). Our analysis suggests that I17, Y305 and A231 might modulate the configuration of t^6^A-catalytic center whereas K202 might participate in tRNA binding and R284 might modulate the function of BUD32.

The precise binding of anticodon stem loop of tRNA to catalytic sites of TsaD/Kae1/Qri7 proteins still remains undetermined and poses an obstacle in mechanistic understanding of KEOPS. Our model of KEOPS–tRNA shows that the connecting loop (residues 36–48) between β1 and α1 of KAE1 adopts a conformation that creates steric clashes with incoming anticodon stem loop of tRNA (Figure 3C). Sequence and structural alignment show that these connecting loops in eukaryotic Kae1/KAE1/OSGEP proteins are highly conserved (Figure 4A) and conformationally divergent (Supplementary Figure S3A). However, the functions of these loops of TsaD/Kae1/Qri7 family proteins remain unexplored. We generated two *At*KEOPS variants with multiple mutations in the loop connecting β1 and α1 of KAE1―K^Y36R/I37K/T38R/P39R/P40R/G41W/H42D^BCP (K^mutant-1^BCP) and K^G43E/F44R/L45K/P46R/R47D/E48K^BCP (K^mutant-2^BCP). We substituted non-charged residues with positively-charged residues of arginine and lysine in these two mutants. In K^mutant-2^BCP, we additionally substituted positively-charged Arg47 with Asp and negatively-charged Glu48 with Lys, respectively. Our purification and SEC analysis demonstrate that these multiple mutations do not affect solubility of KAE1 and overall structure of KEOPS (Supplementary Figure S5A and B). Our gel analysis shows that both K^mutant-1^BCP and K^mutant-2^BCP are capable of binding 5′-6FAM-tRNA^Arg^_CCU_ (Supplementary Figure S5D). Notably, MST measurements demonstrate an enhanced interaction between K^mutant-1^BCP and 5′-6FAM-tRNA^Arg^_CCU_ (*K*_d_= 5.27 μM) but no interaction between K^mutant-2^BCP and 5′-6FAM-tRNA^Arg^_CCU_ (Figure 4C). Both K^mutant-1^BCP and K^mutant-2^BCP lose the t^6^A-catalytic activity (Supplementary Figure S5E, Figure 4D), suggesting that the connecting loop between β1 and α1 of KAE1 is essentially required for tRNA t^6^A biosynthesis by KEOPS. In particular, our model of KEOPS–tRNA and interaction analysis suggest that the first half of the loop (residues 36–42) might participate in tRNA binding (Figure 4B), as K^mutant-1^BCP has acquired increased affinity towards tRNA (Figure 4C).

### KEOPS is modulated by BUD32 via the C-terminal tail and ATP to ADP hydrolysis

**Figure 5.**
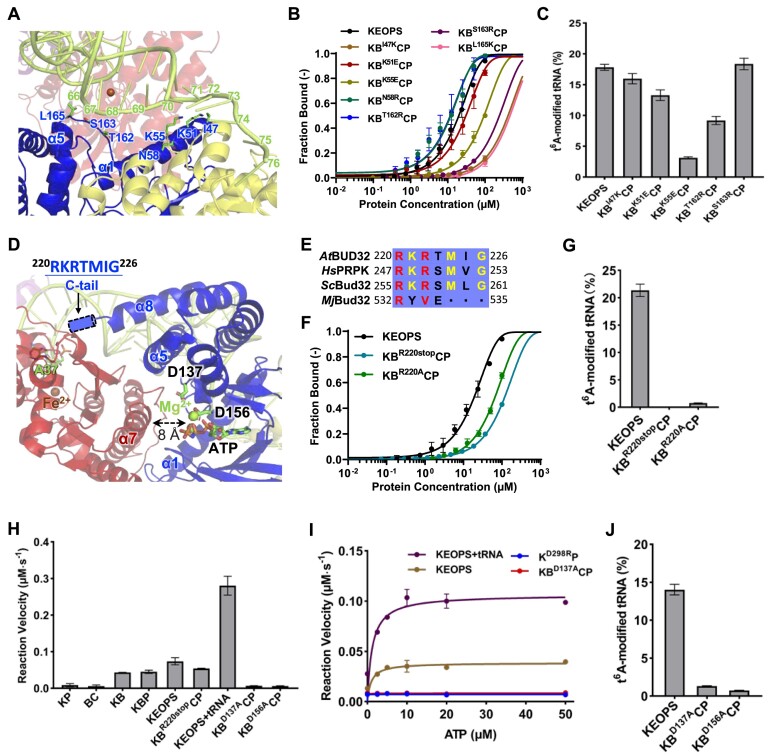
Functional modulation of *A. thaliana* KEOPS by BUD32. (**A**) The model of *A. thaliana* KEOPS–tRNA shows residues of BUD32 (colored in blue) in proximity to amino acid acceptor arm of tRNA. (**B**) Microscale thermophoresis (MST) measurements of the interactions between 50 nM 5′-6FAM-tRNA^Arg^_CCU_ and KEOPS variants bearing mutations in BUD32. (**C**) Comparison of the t^6^A modification efficiencies of IVT *At*tRNA^Arg^_CCU_ by KEOPS variants bearing mutations in BUD32. Error bars represent standard deviations for triplicate measurements. (**D**) The interacting interface of KAE1 (colored in red) and BUD32 (colored in blue) in the model of *A. thaliana* KEOPS–tRNA. ATP and Mg^2+^ ions were projected in the ATPase-catalytic site of BUD32 according to the crystal structures of *H. sapiens* PRPK (PDB: 6WQX) and *S. cerevisiae* Bud32 (PDB: 4WW9). The C-terminal tail (^222^RTMIG^226^) as represented in dashed cylinder was not observed in cryo-EM structure of *A. thaliana* KEOPS complex. tRNA A37 and Fe^2+^ ion indicate catalytic center of KAE1. (**E**) Local sequence alignment of the C-terminal tails of *A. thaliana* (*At*) BUD32, *H. sapiens* (*Hs*) PRPK, *S. cerevisiae* (*Sc*) Bud32 and *M. jannaschii* (*Mj*) Bud32. (**F**) MST measurements of the interactions between 50 nM 5′-6FAM-tRNA^Arg^_CCU_ and *At*KEOPS, *At*KB^R220stop^CP or KB^R220A^CP. Error bars represent standard deviations for triplicate measurements. (**G**) Comparison of the t^6^A modification efficiencies of *A. thaliana* KEOPS, KB^R220stop^CP or KB^R220A^CP towards IVT *At*tRNA^Arg^_CCU_. (**H**) Hydrolysis rate of 2 mM ATP to ADP by 5 μM *A. thaliana* KEOPS complex, subcomplexes or variants in the presence or absence of 10 μM IVT *At*tRNA^Arg^_CCU_. (**I**) Kinetic plots of hydrolysis of ATP to ADP by 2 μM *A. thaliana* KEOPS, K^D298R^P or KB^D137A^CP in the presence or absence of 10 μM IVT *At*tRNA^Arg^_CCU_. (**J**) Comparison of the t^6^A modification efficiencies of *A. thaliana* KEOPS, KB^D137A^CP or KB^D156A^CP towards IVT *At*tRNA^Arg^_CCU_.

Our model of KEOPS–tRNA coupled interaction analysis features an essential role of BUD32 in direct binding of tRNA to KEOPS (Figure 3D, Figure 5A). The model suggests that the 3′ amino acid acceptor arm (nucleobases 66–71) of tRNA is located in close proximity to α1 and α5 of BUD32 (Figure 5A). To confirm the participation of α1 and α5 of BUD32 in tRNA binding, we generated KEOPS variants bearing mutations in α1 (I47K, K51E, K55E and N58R) and in the connecting loop of β8 and α5 (T162R, S163R and L165K) of BUD32. We purified these BUD32-mutated KEOPS variants―KB^I47K^CP, KB^K51E^CP, B^K55E^CP, KB^N58R^CP, KB^T162R^CP, KB^S163R^CP and KB^L165K^CP (Supplementary Figure S6A and B). We first measured the interactions between 5′-6FAM-tRNA^Arg^_CCU_ and KEOPS variants by EMSA and MST. EMSA analysis shows that K55E mutation severely affects the interaction between 5′-6FAM-tRNA^Arg^_CCU_ and KEOPS; K51E, T162R or S163R mutation confers a milder effect; I47K, N58R or L165K mutation exerts no effect (Supplementary Figure S6C). However, quantitative analysis by MST demonstrates that I47K, K55E, S163R, L165K or K51E mutation leads to weaker interaction between 5′-6FAM-tRNA^Arg^_CCU_ and KEOPS while N58R or T162R mutation slightly promotes the binding of 5′-6FAM-tRNA^Arg^_CCU_ to KEOPS (Figure 5B, Table 2). It seems that substitution of Lys55 by negatively-charged Glu leads to markedly weak binding affinity of KEOPS towards 5′-6FAM-tRNA^Arg^_CCU_ whereas substitution of Asn58 and Thr162 by positively-charged Arg enhances the binding affinities, suggesting that Lys55, Asn58 and Thr162 participate in tRNA binding. We then measured the t^6^A-catalytic activities of these KEOPS variants (Supplementary Figure S6D, Figure 5C) and demonstrate that K55E mutation substantially compromises t^6^A-cataalytic activity of KEOPS; K51E or T162R mutation mildly affects the t^6^A-catalytic activity; I47K, N58R, S163R or L165K mutation confers negligible effect (Figure 5C). Nonetheless, K55E mutation causes a drop in both binding affinity and t^6^A-catalytic activity, strongly documenting a role for Lys55 of BUD32 in tRNA binding.

Our cryo-EM structure of *At*KEOPS reveals distinct conformation of the C-lobe of BUD32 in relation to KAE1 compared to that of *Mj*KEOPS. Strikingly, the C-terminal tail of BUD32 adopts a unique conformation (Figure 3C). In both structures, the C-terminal tails of BUD32/Bud32 protrude towards the catalytic site of KAE1/Kae1. However, the structure of the tail sequence (^220^RKRTMIG^226^) was not observed in our structure (Figure 5D) nor in structures of BUD32 orthologs (3,56,57). Sequence alignment reveals that the tail sequences are highly conserved in eukaryotic BUD32/PRPK/Bud32 proteins but less conserved in archaean Bud32, which lacks a MxG motif (Figure 5E). The C-terminal tails of BUD32/PRPK/Bud32 proteins contain a positively-charged RKR motif (Figure 5E). To analyze the function of the C-terminal tail of BUD32, we generated a series of KEOPS variants, including a variant lacking ^220^RKRTMIG^226^ (KB^R220stop^CP) and eight variants with single mutation―KB^R220A^CP, KB^K221A^CP, KB^R222A^CP, KB^T223A^CP, KB^M224A^CP, KB^I225A^CP, KB^G226A^CP and KB^G226R^CP (Figure 5D). We purified good quality proteins of these KEOPS variants (Supplementary Figure S7A and B). Our EMSA analysis shows that the binding of 5′-6FAM-tRNA^Arg^_CCU_ to KEOPS is not essentially affected or disrupted by deletion of ^220^RKRTMIG^226^ or single mutation in it (Supplementary Figure S7C). MST measurement demonstrates that the interaction between 5′-6FAM-tRNA^Arg^_CCU_ and KB^R220A^CP or KB^R220stop^CP was weaker than wild-type KEOPS (Figure 5F). The *K*_d_ values for KEOPS, KB^R220stop^CP and KB^R220A^CP are 18.08 μM, 148.39 μM and 88.20 μM, respectively. We performed tRNA t^6^A assay using IVT *At*tRNA^Arg^_CCU_ and these variants (Supplementary Figure S7D), and detected no t^6^A with KB^R220stop^CP and only trace amount of t^6^A with KB^R220A^CP, respectively (Figure 5G). In contrast, we detected large amount of t^6^A that is comparable to wild-type KEOPS in assays using KB^K221A^CP, KB^M224A^CP, KB^I225A^CP, KB^G226A^CP or KB^G226R^CP. In parallel, we detected decreased levels of t^6^A in assays using KB^R222A^CP or KB^T223A^CP. These data demonstrate that Arg220 of BUD32 plays an essential role in regulating the t^6^A-catalytic activity of KEOPS.

Previous studies demonstrated that Bud32/PRPK regulates the function of KEOPS via an ATP to ADP hydrolysis-based mechanism (20,45). We first measured the ATPase activity of *At*KEOPS complex and subcomplexes using an NADH-coupled ATPase assay. We determined an efficient conversion of ATP to ADP in the presence of KAE1–BUD32, PCC1–KAE1–BUD32 or KEOPS, but neither KAE1–PCC1 nor BUD32–CGI121 (Supplementary Figure S6E, Figure 5H). Comparison of the ATP hydrolysis rates (reaction velocity in steady state) demonstrate that BUD32 catalyzes the ATP hydrolysis and KAE1 stimulates the ATPase activity of BUD32, which is further potentiated by CGI121 (Figure 5H). Structural juxtaposition of *At*BUD32 and *Hs*PRPK in complex with ATP and Mg^2+^ projects ATP and two Mg^2+^ ions in the catalytic site of *At*BUD32 (Figure 5D), which reveals that the conserved D137 and D156 might participate in coordination of ATP and Mg^2+^ (Figure 5D). We mutated D137 and D156 of BUD32 and generated two KEOPS variants―KB^D137A^CP and KB^D156A^CP (Supplementary Figure S6A and B). We show that KB^D137A^CP and KB^D156A^CP is no longer active in hydrolyzing ATP to ADP (Figure 5H). Meanwhile, KB^R220stop^CP exhibits an ATPase activity that is comparable to the KEOPS (Figure 5H), suggesting that the C-terminal tail of BUD32 does not directly participate in ATP hydrolysis. We further determined that IVT *At*tRNA^Arg^_CCU_ markedly potentiates the ATP hydrolysis by KEOPS (Figure 5H). We measured catalytic kinetics of ATP hydrolysis by 2 μM KEOPS (Figure 5I): *V*_max_= 0.039 ± 0.0015 μM·s^–1^, *K*_m_= 0.95 ± 0.053 μM, *K*_cat_= 0.019 ± 0.00073 s^–1^, *K*_cat_/*K*_m_= 0.020 μM^–1^·s^–1^. In parallel, we determined catalytic kinetics of 2 μM KEOPS in the presence of 10 μM IVT *At*tRNA^Arg^_CCU_: *V*_max_= 0.11 ± 0.00021 μM·s^–1^, *K*_m_= 1.22 ± 0.029 μM, *K*_cat_= 0.053 ± 0.00011 s^–1^, *K*_cat_/*K*_m_= 0.043 μM^–1^·s^–1^. The kinetic parameters indicate that tRNA potentiates hydrolysis of ATP to ADP by KEOPS but slightly reduces the binding affinity of ATP to KEOPS. Thus, it suggests that binding of tRNA to KEOPS directly induces a conformational change in the catalytic site of BUD32. We further determined that either KB^D137A^CP or KB^D156A^CP still sustains a very low level of t^6^A-catalytic activity (Supplementary Figure S6D and Figure 5J), suggesting that deprivation of the ATPase activity of BUD32 severely interferes with turnover of t^6^A-catalysis by KEOPS.

### Proteomic analysis of KEOPS–interacting proteins

Recently, Daugeron *et al.* identified no orthologs of Gon7/Pcc2 in *Arabidopsis thaliana* using advanced comparative genomic analysis (46). Our *in vitro* assay also confirmed a t^6^A-catalytic activity of *At*KEOPS on IVT tRNAs. Nonetheless, we performed proteomic analysis of KEOPS-interacting proteins using LC–MS/MS. We simply incubated purified KEOPS with total soluble proteins isolated from *Arabidopsis thaliana* seedlings and purified potential interactors of 6His tagged KEOPS by Ni-NTA chromatography. Unbound proteins were removed by repeated washing using lysis buffer until no proteins appeared in the wash fraction prior to final elution of KEOPS. SDS–PAGE analysis showed that the final elution sample contained four subunits of KEOPS and very weak bands of proteins (data not shown). The final elution sample were digested into tryptic peptides and applied to LC–MS/MS for proteomic analysis. In total, 671 proteins were identified in the MS samples and listed in order according to the intensity-based absolute quantification (iBAQ) values (Supplementary Table S4). The list contains 664 characterized proteins with functions implicated in a wide range of cellular processes and 7 uncharacterized proteins (Uniprot IDs: Q9FKA5, A0A1P8B3M2, Q9C6U3, O64818, Q9FZG2, Q93W28 and F4J0L7). Notably, Q9FZG2 has a molecular size of 10.67 kDa, which is close to that of GON7/Gon7. Interestingly, AlphaFold prediction structure of Q9FZG2 (entry: Q9FZG2, www.alphafold.ebi.ac.uk) shows that Q9FZG2 is an intrinsically disordered protein with a structured core comprising two antiparallel β-stands connected to an α-helix.

## Discussion

Evolutionarily conserved KEOPS catalyzes the transfer of TC-moiety from TC-AMP to N6 atom of adenine at position 37 of tRNA, leading to tRNA t^6^A. Up to date, characterizations of KEOPSs (*Methanococcus jannaschii*, *Saccharomyces cerevisiae* and *Homo sapiens*) revealed structures, overall arrangement and functions of KEOPS subunits (31). However, the structure of a complete KEOPS and molecular workings of KEOPS still remain unresolved. In the present study, we determined a cryo-EM structure of KEOPS complex from *Arabidopsis thaliana* and performed structure–function relationship analysis of *A. thaliana* KEOPS. The data analysis and findings are discussed below.

We successfully purified recombinant KEOPS proteins and reconstituted a stable KEOPS complex that is composed of KAE1, BUD32, CGI121 and PCC1. We show that the four-subunit *A. thaliana* KEOPS is active in catalyzing tRNA t^6^A biosynthesis in conjunction with recombinant *A. thaliana* YRDC (Figure 1C). Though no ortholog of GON7/Gon7 in *Arabidopsis thaliana* has been identified using bioinformatic analysis (46), we identified a large number of potential KEOPS interactors using pull-down assay and LC–MS/MS proteomic analysis (Supplementary Table S4). Of note, the uncharacterized Q9FZG2 (At1g47820) and O64818 (At2g23090) possess GON7/Gon7 features―comparable molecular sizes and partial intrinsically disordered structures. The structures and functions of Q9FZG2 and O64818 remain to be experimentally determined. Moreover, the presence of other proteins in MS sample eluted with KEOPS suggests that KEOPS or subunits might interact weakly or transiently with identified proteins. However, such a hypothesis remains to be experimentally validated in future.

A number of studies demonstrated large variations in t^6^A modification frequencies of KEOPS towards different tRNA substrates (20,44,45,55). Our *in vitro* assay shows that the t^6^A-catalytic activities of KEOPS towards IVT tRNAs are different (Figure 1F). KEOPS exhibits very low t^6^A-catalytic activity on tRNA^Ser^_GCU_ and no activity on tRNA^Asn^_GUU_, tRNA^Met^_CAU_ and tRNA^Ile^_UAU_, of which the latter two substrates lack a 36-UAA-38 motif. Introduction of a 36-UAA-38 motif into tRNA^Ile^_UAU_ restores the t^6^A modification by KEOPS (Figure 1F). As per the inactivity of tRNA^Asn^_GUU_ and low activity tRNA^Ser^_GCU_, other native modifications might be needed to stabilize the active conformations of these two tRNAs for more efficient t^6^A modification. The other explanation is that the *in vitro* folding of IVT tRNAs does not adopt a t^6^A-productive conformation, though our SEC, CD and interaction analysis shows that these IVT tRNAs adopt comparable overall 3D structures.

We determined a *K*_d_ value of 18 μM for the binding of KEOPS with IVT tRNA^Arg^_CCU_ and attempted to determine a cryo-EM structure of KEOPS–tRNA^Arg^_CCU_. Unfortunately, it turned out that tRNA was absent in the cryo-EM structure. Moreover, BUD32–CGI121 of one protomer of the KEOPS dimer was not observed in the structure (Figure 2A). We presume that the interaction between KEOPS and tRNA^Arg^_CCU_ was too weak to form a stable complex in the EM sample, as we were not able to purify a KEOPS–tRNA^Arg^_CCU_ by SEC. In another scenario, a fraction of tRNA^Arg^_CCU_ bound to BUD32–CGI121 that was dissociated from KEOPS in the EM sample, as the *K*_d_ for the interaction between BUD32–CGI12 and tRNA^Arg^_CCU_ is 62 μM. Our cryo-EM structure of *At*KEOPS complex reveals a conserved overall arrangement of KEOPS subunits (31). The model of *Mj*KEOPS–tRNA provides a good framework for mechanistic understanding of KEOPS (45). It shows that tRNA binds to an extended surface of KEOPS and makes contacts with the four subunits (31,45). We generated a rigid model for *A. thaliana* KEOPS–tRNA by means of structural juxtaposition of *At*KEOPS and *Mj*KEOPS–tRNA (Figure 2E). We further manually refined the geometric and electrostatic fitting of tRNA to KEOPS with reference to conformation of the C-lobes of *Mj*Bud32/*At*BUD32, which is directly involved in tRNA binding (45). The resulting model of KEOPS–tRNA reveals a good geometric complementarity and electrostatic interaction (Figure 3D). The model features a central role of BUD32 in tRNA binding. Our mutational analysis of BUD32 supports a direct participation of α1, on which the K55E mutation leads to markedly weak interaction between KEOPS and tRNA^Arg^_CCU_ (Figure 5B) and decreased t^6^A-catalytic activity of KEOPS (Figure 5B). In addition, a number of other mutations in the connecting loop of β8 and α5 of BUD32 also interferes with the interaction between KEOPS and tRNA^Arg^_CCU_. Moreover, we show that the loop connecting β1 and α1 of KAE1 participates in binding of tRNA to KEOPS (Figure 4B). We determined that CGI121 or PCC1–KAE1 is not capable of binding tRNA but BUD32–CGI121 and KAE1–BUD32–PCC1 can independently bind tRNA (Figure 3A and B). Subtraction of CGI121 from KEOPS or deletion of 3′ CCA end of tRNA^Arg^_CCU_ only reduces the t^6^A-catalytic activity of KEOPS (Figure 1H). In contrast, *Mj*Cgi121 alone or *Mj*Bud32–Cgi121 is capable of binding tRNA but *Mj*Kae1–Bud32–Pcc1 does not bind tRNAs (45). *Pa*Pcc1–Kae1 is a binding core for tRNAs and *Pa*Cgi121 alone does not bind tRNAs (20). The functional differences among different KEOPS proteins imply specific mechanisms underscoring the molecular interaction and regulation of KEOPS–tRNA assembly. However, mutational analysis of the interactions and t^6^A-catalytic activities justifies at least the binding orientation of tRNA to KEOPS in our model of *At*KEOPS–tRNA.

Our cryo-EM structure of *A. thaliana* KEOPS revealed a dimeric structure of KEOPS, which is consistent with the dimeric state of *At*KEOPS as determined by SEC. *A*tKEOPS dimer is mediated by homodimerization of PCC1 in a similar manner as for archaean KEOPS (Supplementary Figure S3E) (44). We generated a four-subunit KEOPS monomer (P^R70D/R73D/A74E^KBC) via disrupting the dimerization of PCC1. KEOPS monomer is capable of binding tRNA (Figure 3F). However, the binding affinity for KEOPS monomer and tRNA^Arg^_CCU_ (*K*_d_= 74 μM) is close to that for BUD32–CGI121 and tRNA^Arg^_CCU_ (*K*_d_= 62 μM), which are greatly lower than the binding affinity of wild-type KEOPS dimer towards tRNA^Arg^_CCU_ (Table 2). Nonetheless, KEOPS monomer is no longer active in biosynthesizing tRNA t^6^A (Figure 3G). Our model of *At*KEOPS–tRNA shows that one KEOPS protomer could binds one tRNA, whose anticodon stem loop potentially makes contacts with PCC1–KAE1 from the other protomer (Figure 2E, Figure 3D). Therefore, we presume that correct binding of tRNA to one KEOPS protomer is facilitated by the other protomer. However, we cannot exclude the possibility that KEOPS dimer binds only one molecule of tRNA. In this case, binding of tRNA to one protomer precludes the binding of second tRNA to the other protomer. Such a similar mechanism is adopted by mitochondrial Qri7 dimer (34) and bacterial TsaD2–TsaB2 tetramer (38). Unfortunately, we could not determine the stoichiometry and number of binding sites by MST and EMSA measurements. In sum, our data shows that *At*KEOPS dimer is required to form a t^6^A-catalytic KEOPS–tRNA assembly.

ATP to ADP hydrolysis by BUD32 is involved in the turnover of t^6^A-catalysis by KEOPS. Such a mechanism is also adopted by bacterial TsaDBE complex (38,41). The ATPase catalytic site is sandwiched between the N-lobe and C-lobe of Bud32/PRPK (56,57). We show that ATP to ADP hydrolysis by BUD32 is stimulated by KAE1 and further strongly potentiated by tRNA^Arg^_CCU_ (Figure 5H). Deletion of C-terminal tail or abrogation of ATPase activity of *At*BUD32 does not interfere with binding of tRNA to *At*KEOPS but leads to dead t^6^A-catalytic activity. But how the C-terminal tail and ATPase activity of BUD32 affect the t^6^A activity of KEOPS remains a long-standing question. Here we determined that Arg220 at the C-terminal tail of BUD32 is critical for the t^6^A-catalytic activity of Kae1. Our structure shows a distance of ∽25 Å between Arg220 of BUD32 and t^6^A-catalytic site of KAE1. Therefore, it's unlikely that Arg220 of BUD32 reaches into the t^6^A-catalytic site of KAE1 and modulates the TC-transfer reaction. We presume that Arg220 or the positively charged C-terminal tail of *At*BUD32 might play a pivotal role in stabilizing anticodon stem loop of tRNA. In structure of *At*KEOPS, the closest distance between α7 of KAE1 and ATPase catalytic site of BUD32 is less than 8 Å. We hypothesize that α7 of KAE1 interacts with α1 and α5 of BUD32 and stimulates the ATPase activity of BUD32. In turn, the hydrolysis of ATP to ADP drives the relocation of α7 of KAE1. By doing so, the two lobes of BUD32 undergo substantial conformational changes between the ATP-bound state and ADP-bound state. Movement of the C-terminal tail along with the relocation of the C-lobe of BUD32 might participate in binding and release of tRNA. Therefore, the C-terminal tail of BUD32 offers an opportunity to couple ATP hydrolysis with correct binding of tRNA to KEOPS, conferring an allosteric regulation of t^6^A-catalytic cycle of KEOPS.

## Conclusion

We have determined a conserved role of *A. thaliana* KEOPS in tRNA t^6^A biosynthesis. Our structure–function relationship analysis of KEOPS–tRNA assembly suggests that the four-subunit KEOPS dimerize via PCC1 in support of a correct binding of tRNA to KEOPS, which requires the essential contribution of BUD32 and is further promoted by CGI121. BUD32 seems to be key regulator of KEOPS. The C-terminal tail of BUD32 functions as a ‘trigger’ to modulate the t^6^A-catalytic activity of KAE1. KAE1 stimulates the ATPase activity of BUD32 in exchange for turnover of the t^6^A-catalysis, which is driven by ATP to ADP hydrolysis by BUD32. Still, there are several fundamental questions to be addressed in order to mechanistically understand the inner workings and cellular roles of KEOPS machineries. In particular, the specific recognition of ANN-decoding tRNAs by KEOPSs and the chemistry of t^6^A catalysis by Kae1/KAE1/OSGEP await to be fully elucidated. A high-resolution structure of a complete KEOPS–tRNA complex is undoubtedly desirable to solve these mysteries.

## Supplementary Material

gkae179_Supplemental_Files

## Data Availability

The cryo-EM electron density map and the atomic coordinates of *A. thaliana* KEOPS model have been deposited in the Protein Data Bank (https://www.wwpdb.org) under the EMDB ID: EMD-36808 and PDB ID: 8K20.
