# Supplementary material for: Molecular basis of A. thaliana KEOPS complex in biosynthesizing tRNA t6A
Source: Nucleic Acids Res. 2024 Mar 13;52(8):4523–40. doi: 10.1093/nar/gkae179 (PMC11077089; doi:10.1093/nar/gkae179)
Supplement: gkae179_Supplemental_Files [file gkae179_supplemental_files.zip › NAR-03581-R-2023-Supplementary.pdf]

Supplementary Tables and Figures

To

Molecular basis of *A. thaliana* KEOPS complex in biosynthesizing tRNA t^6A

Xinxing Zheng, Chenchen Su, Lei Duan, Mengqi Jin, Yongtao Sun, Li Zhu* and Wenhua Zhang*

School of Life Sciences, Key Laboratory of Cell Activities and Stress Adaptation of the Ministry of Education, Lanzhou University, Lanzhou, 730000, China

* To whom correspondence should be addressed (W.Z.). Tel/Fax: +86-931-8914381; Email: zwh@lzu.edu.cn. Correspondence may also be addressed to zhuli@lzu.edu.cn (L.Z.).

Supplementary Table 1. Primers used for site-directed mutagenesis.

Construct	Forward primer	Reverse primer
**KP$^{R70D/R73D/A74E}$**	AGATCTGCTGGATGAAAGCTTTAGCGCATTTGTTGATGTG	TCATCCAGCAGATCTGCTTCAATCGCTTCAAAATGAAC
**KP^{R73D}**	TCTGCTGGATGCAAGCTTTAGCGCATTTGTTGATG	CTTGCATCCAGCAGACGTGCTTCAATCGC
**K^{H117A}P**	TGTGGCTGCAATTGAGATGGGGAGAGTAGTTACTG	TCAATTGCAGCCACACAATGATTCACAGC
**K^{D298R}P**	CTGCATCCGAAATGGAGCTATGATCGCTTACACAG	CCATTTCGGATGCAGTAACGGTCATCTGTTGC
**K$^{mutant\text{-}1}$P**	TAAAAGACGACGTTGGGATGGTTTTCTTCCTAGAGAGACTGC	CAACGTCGTCTTTTACGCGTATGCCGAGGATTTGC
**K$^{mutant\text{-}2}$P**	AACGTAAACGTGATAAGACTGCACATCACCATCTTGACCA	TATCACGTTTACGTTCATGACCAGGTGGTGTTATATACGT
**K^{A231G}P**	TACACCAGGAGACTTGTGTTATTCCCTTCAAGAG	AAGTCTCCTGGTGTACACTCGTTATTCTTGAGC
**K^{Y305A}P**	GATCGCTGCCACAGGTCTACTCGCGTTTG	CCTGTGGCAGCGATCATAGCTCCATTATCG
**K^{I17F}P**	TAACAAGTTTGGAGTTGGAATTGTGACTTTAGACG	ACTCCAAACTTGTTAGCTGATCCTTCGAAACC
**K^{K202R}P**	TGCTGTTAGAGGTATGGATGTATCGTTCAGCGG	ATACCTCTAACAGCATACGGAAGATCAATAAAG
**K^{R284C}P**	CTCAGAATGTGATGGTAAGCTGTTTGCAACA	CCATCACATTCTGAGCACATAGTCCTCATCA
**B^{D137A}C**	GCATGGTGCACTGACGACCTCAAATATGCTGGTTC	GTCAGTGCACCATGCGCCAGACCACC
**B^{D156A}C**	TCTGATTGCATTTGGTCTGAGCGTTACCAGTACG	CCAAATGCAATCAGAACCAGCTGATTGGTACC
**B^{R220stop}C**	AGCGTGGTTAACTAGATCTAGAATTTTGTTTAA	CTAGTTAACCACGCTGACGAACCTGT
**B^{R220A}C**	GCGTGGTGCAAAACGCACAATGATTGGCTAAC	CGTTTTGCACCACGCTGACGAACCTG
**B^{K221A}C**	TGGTCGTGCACGCACAATGATTGGCTAACTAG	GTGCGTGCACGACCACGCTGACGAAC
**B^{R222A}C**	TCGTAAAGCAACAATGATTGGCTAACTAGATCTAG	ATTGTTGCTTTACGACCACGCTGACGA
**B^{T223A}C**	TAAACGCGCAATGATTGGCTAACTAGATCTAG	ATCATTGCGCGTTTACGACCACGCTGAC
**B^{M224A}C**	ACGCACAGCAATTGGCTAACTAGATCTAG	CCAATTGCTGTGCGTTTACGACCACG
**B^{I225A}C**	CACAATGGCAGGCTAACTAGATCTAGAATTTTGTT	TAGCCTGCCATTGTGCGTTTACGACCACG
**B^{G226A}C**	AATGATTGCATAACTAGATCTAGAATTTTGTTTAA	AGTTATGCAATCATTGTGCGTTTACGA
**B^{G226R}C**	AATGATTCGTTAACTAGATCTAGAATTTTGTTTAA	AGTTAACGAATCATTGTGCGTTTACGA
**B^{I47K}C**	TCATCCTAAACTGGATGCAAAACTGACCCTG	TCCAGTTTAGGATGACGATACTTTTTGCTAA
**B^{K51E}C**	GGATGCAGAACTGACCCTGAAACGTCTGAATGC	GTCAGTTCTGCATCCAGAATAGGATGACG
**B^{L54K}C**	ACTGACCAAAAAACGTCTGAATGCAGAAGCACG	CGTTTTTTGGTCAGTTTTGCATCCAGAATAGG
**B^{K55E}C**	GACCCTGGAACGTCTGAATGCAGAAGCACGC	AGACGTTCCAGGGTCAGTTTTGCATCCAG
**B^{N58R}C**	ACGTCTGAGAGCAGAAGCACGCTGTATGACC	TCTGCTCTCAGACGTTTCAGGGTCAGTTTTGC
**B^{T162R}C**	GAGCGTTAGAAGTACGCTGCCGGAAGATAAAG	GTACTTCTAACGCTCAGACCAAAATCAATCAG
**B^{S163R}C**	CGTTACCAGAACGCTGCCGGAAGATAAAGC	AGCGTTCTGGTAACGCTCAGACCAAAATC
**B^{L165K}C**	CAGTACGAAGCCGGAAGATAAAGCAGTTGATCTG	TCCGGCTTCGTACTGGTAACGCTCAGACC

Supplementary Table 2. Primers of DNA templates for *in vitro* transcription of tRNAs.

	Forward primer	Reverse primer
**$tRNA^{Thr}_{CGU}$**	TAATACGACTCACTATAGCCTCCGTAGCATAGTGGTATT GCGTTCGCTTCG	TGGAGCCTACGGTGAGGATCGAACTCACGACCTTTCGC TTACGAAGCGAAC
**$tRNA^{Asn}_{GUU}$**	TAATACGACTCACTATAGCTGGAATAGCTCAGTAGGTTT AGAGCGTGTGGCTGT	TGGCGCTGAAAGGAGGGGTCGAACCTCCGACCTTGAG GTTAACAGCCACACGCT
**$tRNA^{Met}_{CAU}$**	TAATACGACTCACTATAGGGGTGGTGGCGCAGTTGGCT AGCGCGTAGGTCTCATAGCTA	TGGTGGGGTGAGAGAGGCTCGAACTCTCGACCTCAGG ATCACTCTGTAGCTATGAGACC
**$tRNA^{Arg}_{UCU}$**	TAATACGACTCACTATAGCGCCCGTGGCCTAATGGATAA GGCGTTTGACTTCTAATCAA	TGGCACGCCCGGTGGGACTCGAACCCACAATCGTTTGA TTAGAAGTCAAACGCCTTATCC
**$tRNA^{Arg}_{CCU}$**	TAATACGACTCACTATAGCGCCTGTAGCTCAGTGGATAG AGCGTCTGTTTCCTAAGCAG	TGGCGCGCCAGGTAGGGGTCGAACCTACGACCTTCTGC TTAGGAAACAGACGCTCTATC
**$tRNA^{Arg}_{CCU}$-ΔCCA**	TAATACGACTCACTATAGCGCCTGTAGCTCAGTGGATAG AGCGTCTGTTTCCTAAGCAG	CGCGCCAGGTAGGGGTCGAACCTACGACCTTCTGCTTA GGAAACAGACGCTCTATC
**$tRNA^{Lys}_{UUU}$**	TAATACGACTCACTATAGCCGTCTTAGCTCAGCGGTAGA GCGCGTGGCTTTTAACCACG	TGGCGCCGTCTGTGGGGATCGAACCCACGGCCACGTG GTTAAAAGCC
**$tRNA^{Ile}_{AAU}$**	TAATACGACTCACTATAGGCCTATTAGCTCAGTTGGTTA GAGCGTCGTGCTAAT	TGGTGGCCTATACAGGGATCGAACCTGTGACCTTCGCG TTATTAGCACGACGCTC
**$tRNA^{Ile}_{UAU}$**	TAATACGACTCACTATAGCTCCCGTAGCTCAGTTGGTTA GAGCGTTGGTCTTATGAGCC	TGGTGCTTCCGGCGGGGCTCGAACCCGCGACCTTCGG CTCATAAGACCA
**$tRNA^{Ser}_{GCU}$**	TAATACGACTCACTATAGTCGCTTTGGCCGAGTGGTTAA GGCGTGTTCCTGCTAAGTACATGGGGT	TGGCGTCGCCTGACAGATTCGAACTCTCGCGGGGAAAC CCCATGTACTTAGCAGGAACACGCCTTA
**$tRNA^{Ile}_{UAU}$-G37A**	TAATACGACTCACTATAGCTCCCGTAGCTCAGTTGGTTA GAGCGTTGGTCTTATAAGCC	TGGTGCTTCCGGCGGGGCTCGAACCCGCGACCTTCGG CTTATAAGACCA

Supplementary Table 3. Cryo-EM data collection, processing, refinement and model validation statistics.

***A. thaliana* KEOPS**	
**PDB ID/EMDB ID**	**8K20/EMD-36808**
**Data collection**	
Electron microscope	Thermo Fisher Titan Krios G3i
Camera	Gatan Bioquantum K3
Magnification	x105,000
Pixel size (Å/pix)	0.43
Defocus range (μm)	–0.9 to –2.7
Exposure time (s)	3.2
Movie fractions (no.)	40
Total electron dose (e^-/Å^2)	52 to 64
Total micrographs (no.)	14622
**Reconstruction**	
Software	cryoSPARC v3.3
Particles for 2D classification	736,244
Particles for 3D classification	327,681
Particles in the final map (no.)	232,232
Symmetry	C1
Final resolution (Å)	3.2 to 5.5
FSC threshold	0.143
**Model Building and validation**	
Residues	1209
MolProbity score	1.81
Clash score	14.00
R.M.S.D. of bond lengths (Å)	0.25
R.M.S.D. of angles (°)	0.50
**Ramachandran plot**	
Favored (%)	1163 (97%)
Allowed (%)	34 (3%)
Outliers (%)	0

Supplementary Figure 1

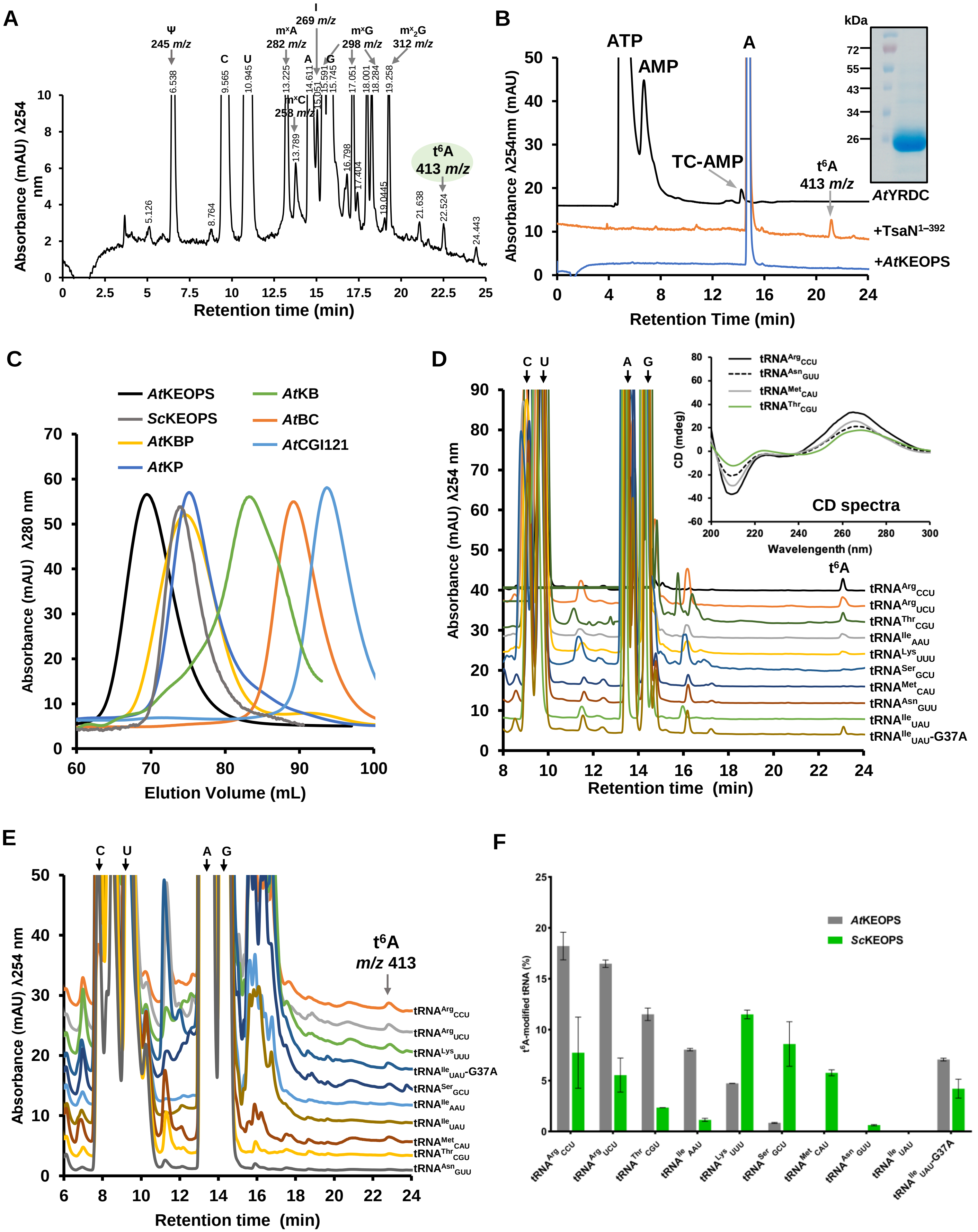

Supplementary Figure 1. *In vitro* enzymatic reconstitution of tRNA t^6A biosynthesis using purified YRDC and KEOPS complex from *Arabidopsis thaliana (At)*. **A.** LC–MS analysis of t^6A and other native modifications that include Pseudouridine (Ψ), Inosine (I), m^xA, m^xC, m^xG and $m^x{}_2G$ (^{x} denotes the unknown atom position of methyl group) in bulk tRNAs isolated from *Arabidopsis thaliana* seedlings. **B.** LC–MS analysis of TC-AMP formation in assay that contained 2 μM *At*YRDC, 4 mM *L*-threonine, 20 mM $NaHCO_3$ and 2 mM ATP; LC–MS analysis of t^6A precursors (t^6ATP/t^6ADP) in TC-AMP assay that was supplemented with 2 μM *P. salinus* $TsaN^{1–392}$ or 2 μM *At*KEOPS. The t^6ATP/t^6ADP assay mixture was first dephosphorylated with alkaline phosphatse and then applied for LC–MS analysis. Purified *At*YRDC is visualized on SDS–PAGE. **C.** Size exclusion chropmatography (SEC) profiles of *A. thaliana* KEOPS (theoretic molecular weight: 94.2 kDa), KAE1–BUD32–PCC1 (KBP) (theoretic molecular weight: 74.6 kDa), KAE1–BUD32 (KB) (theoretic molecular weight (MW): 63.9 kDa), KAE1–PCC1 (KP) (theoretic MW: 49.5 kDa), BUD32–CGI121 (BC) (theoretic MW: 43.9 kDa), CGI121 (theoretic MW: 18.8 kDa) and referenced *S. cerevisiae* KEOPS (five-subunit KEOPS complex with a theoretic MW of 117.7 kDa) using a gel-filtration column (HiLoad 16/600 Superdex 200, GE Healthcare). **D.** HPLC chromatograms of nucleosides of the digested *At*tRNAs that were re-purified from enzymatic assays using 2 μM *At*YRDC, 2 μM *At*KEOPS and 20 μM *in vitro* transcribed (IVT) tRNAs. The chemical identities of A, U, C, G and t^6A were confirmed by mass spectrometer coupled to HPLC. The insert shows the circular dichroism (CD) spectra of folded IVT $tRNA^{Arg}{}_{CCU}$, $tRNA^{Asn}{}_{GUU}$, $tRNA^{Met}{}_{CAU}$ and $tRNA^{Thr}{}_{CGU}$. **E.** HPLC chromatograms of nucleosides of the digested *At*tRNAs from enzymatic assays using 2 μM *At*YRDC, 2 μM *Sc*KEOPS and 20 μM IVT tRNAs of *Arabidopsis thaliana*. **F.** Quantification of the t^6A modification efficiencies by *Sc*KEOPS towards these different IVT tRNAs as measured in (**E**) and comparison of t^6A modification efficiency of *At*KEOPS and *Sc*KEOPS towards the same IVT *At*tRNAs.

Supplementary Figure 2

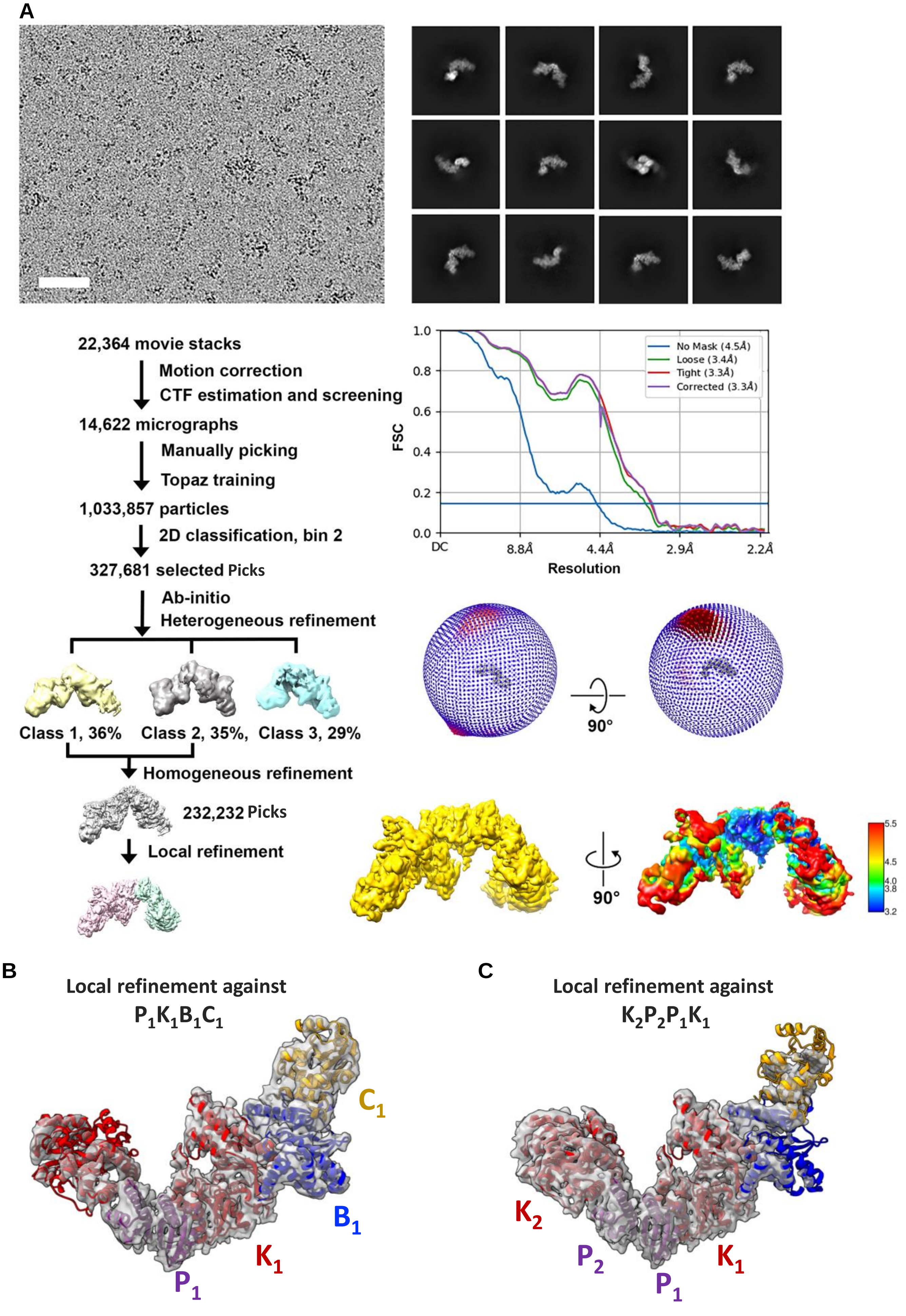

Supplementary Figure 2. Classification, reconstruction and refinement of the cryo-EM maps for *A. thaliana* KEOPS. **A.** Representative cryo-EM micrograph of KEOPS embedded in vitreous ice (scale bar 50 nm), 2D class sorting, flow chart and views of 3D construction, gold-standard Fourier Shell Correlation (FSC) curve, euler angle distribution map of all particles and the resolution landscape of the map for final 3D reconstruction. **B.** and **C.** Local refinement against PCC1–KAE1–BUD32–CGI121 ($P_1K_1B_1C_1$) (**B**) and KAE1–PCC1–PCC1–KAE1 ($K_2P_2P_1K_1$) (**C**).

Supplementary Figure 3

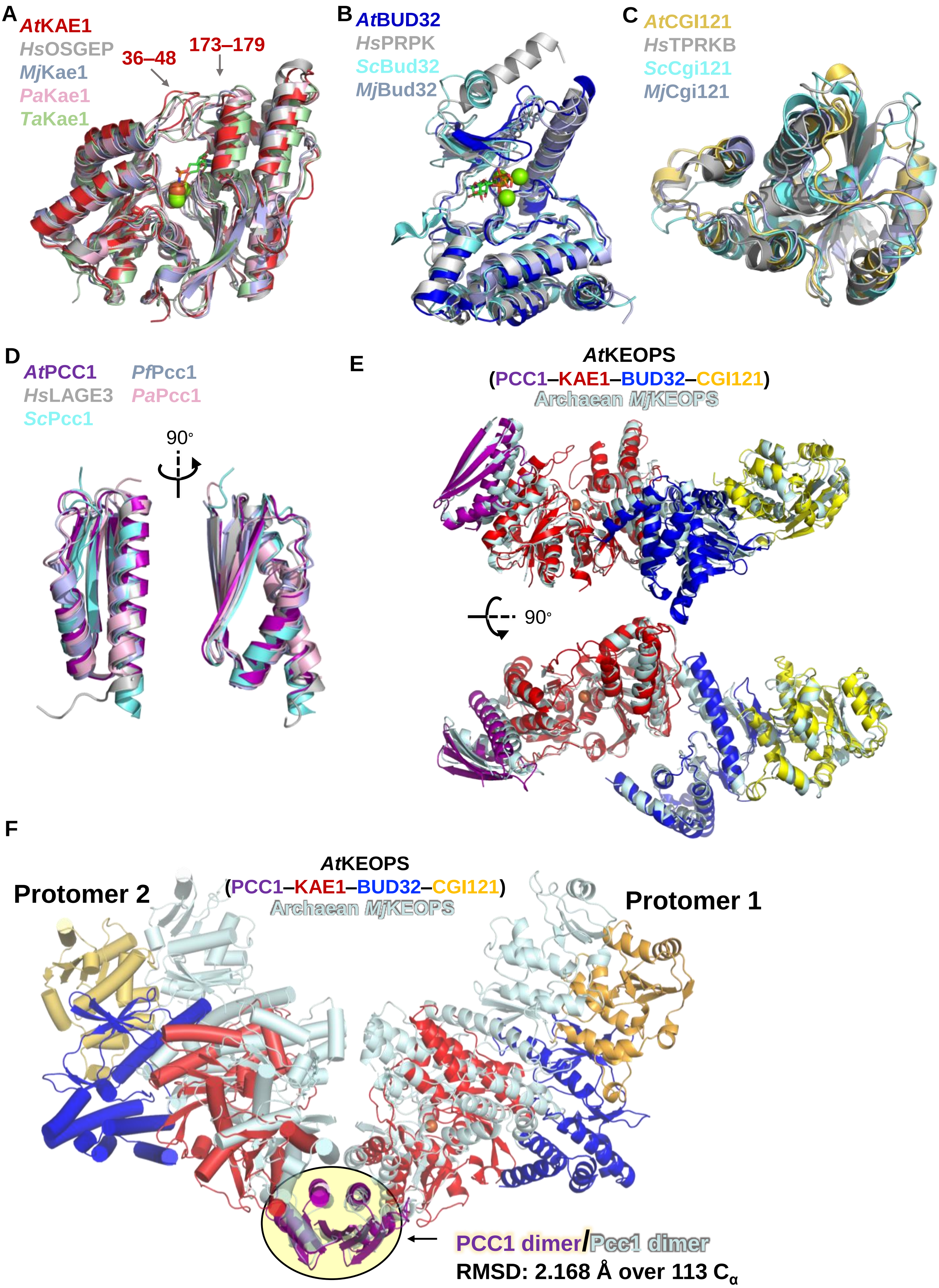

Supplementary Figure 3. Structural comparison of KEOPS proteins from *Arabidopsis thaliana* (*At*), *Homo sapiens* (*Hs*), *Methanocaldococcus jannaschii* (*Mj*), *Pyrococcus abyssi* (*Pa*), *Thermoplasma acidophilum* (*Ta*), *Pyrococcus furiosus* (*Pf*) and *Saccharomyces cerevisiae* (*Sc*). **A.** Juxtaposition of cryo-EM structure of *At*KAE1 and crystal structures of *Hs*OSGEP (PDB: 6GWJ), *Mj*Kae1 (PDB: 5JMV), *Pa*Kae1 (PDB: 2IVN) and *Ta*Kae1 (PDB: 3ENO). Two flexible loops (36–48 and 173–179) atop the catalytic site of *At*KAE1 are indicated. **B.** Juxtaposition of cryo-EM structure of *At*BUD32 and crystal structures of *Hs*PRPK (PDB: 6WQX), *Sc*Bud32 (PDB: 4WW9) and *Mj*Bud32 (PDB: 3ENH). **C.** Juxtaposition of cryo-EM structure of *At*CGI121 and crystal structures of *Hs*TPRKB (PDB: 6WQX), *Sc*Cgi121 (PDB: 4WW9) and *Mj*Cgi121 (PDB: 3ENH). **D.** Juxtaposition of cryo–EM structure of *At*PCC1 and crystal structures of *Hs*LAGE3 (PDB: 6GWJ), *Sc*Pcc1 (PDB: 4WXA), *Pf*Pcc1 (PDB: 5JMV) and *Pa*Pcc1 (PDB: 7A67). **E.** Alignment of crystal structures of *Pf*Pcc1–*Mj*Kae1 and *Mj*Bud32–*Mj*Cgi121 to their counterparts in the cryo-EM structure of *At*KEOPS, which gives an RMSD of 1.96 Å (over 380 C_α atoms) and 2.95 Å (over 320 C_α atoms), respectively. **F.** Structrual comparison of archaean KEOPS dimer and *At*KEOPS dimer by means of superposing *At*PCC1 dimer and *Pf*Pcc1 dimer, which gives an RMSD of 2.168 Å over 113 C_α atoms.

Supplementary Figure 4

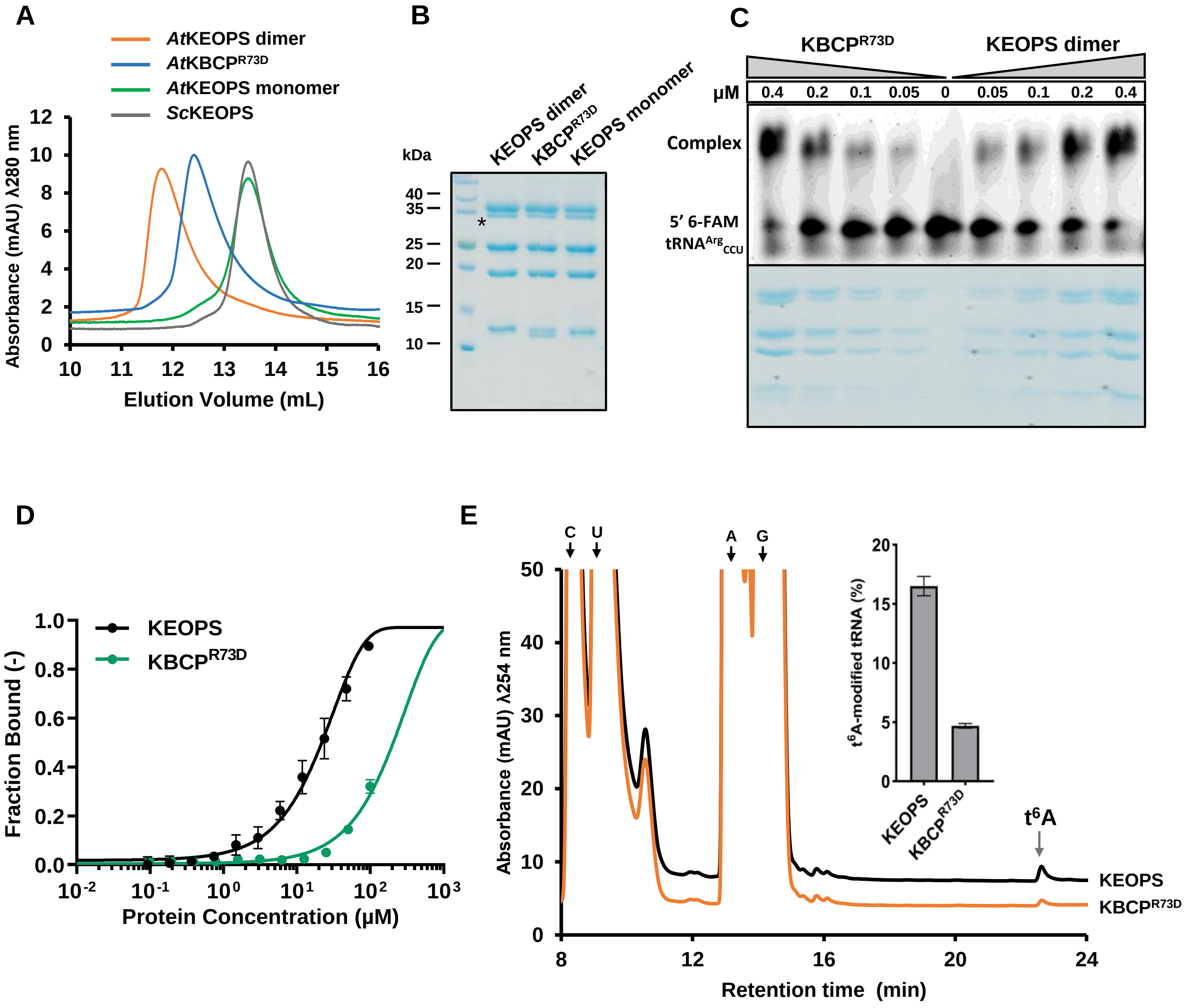

Supplementary Figure 4. Biochemical and functional characterization of *At*KEOPS variant—KAE1–BUD32–CGI121–PCC1^{R73D} (KBCP^{R73D}). **A.** SEC (Superdex 200 Increase 10/300 GL, GE Healthcare) profiles of KBCP^{R73D}, wild-type KEOPS (KEOPS dimer), KBCP$^{R70D/R73D/A74E}$ (KEOPS monomer) and referenced *Sc*KEOPS (five-subunit KEOPS monomer). **B.** SDS–PAGE analysis of *At*KEOPS, KBCP^{R73D} and KBCP$^{R70D/R73D/A74E}$ which were applied to SEC analysis in (**A**). * indicates KAE1 degradation (confirmed by LC–MS/MS). **C.** EMSA analysis of the interactions between 0.1 μM 5'-6FAM-tRNA$^{Arg}_{CCU}$ and 0.05–0.4 μM *At*KBCP^{R73D} or *At*KEOPS. The migration of 5'-6FAM-tRNA$^{Arg}_{CCU}$ is shown in th upper gel, and 10-fold amount of the original protein input in each lane are visualized on a separate SDS–PAGE in the lower panel. **D.** Microscale thermophoresis (MST) analysis of the interactions between 50 nM 5'-6FAM-tRNA$^{Arg}_{CCU}$ and 97 nM–100 μM *At*KBCP^{R73D} (K_d≈372 μM) or *At*KEOPS dimer (K_d≈18 μM). **E.** LC–MS analysis of the t^{6}A-catalytic activity of KBCP^{R73D} on IVT *At*tRNA$^{Arg}_{CCU}$ and the comparison of the t^{6}A modification efficiencies of *At*KEOPS dimer and KBCP^{R73D}.

Supplementary Figure 5

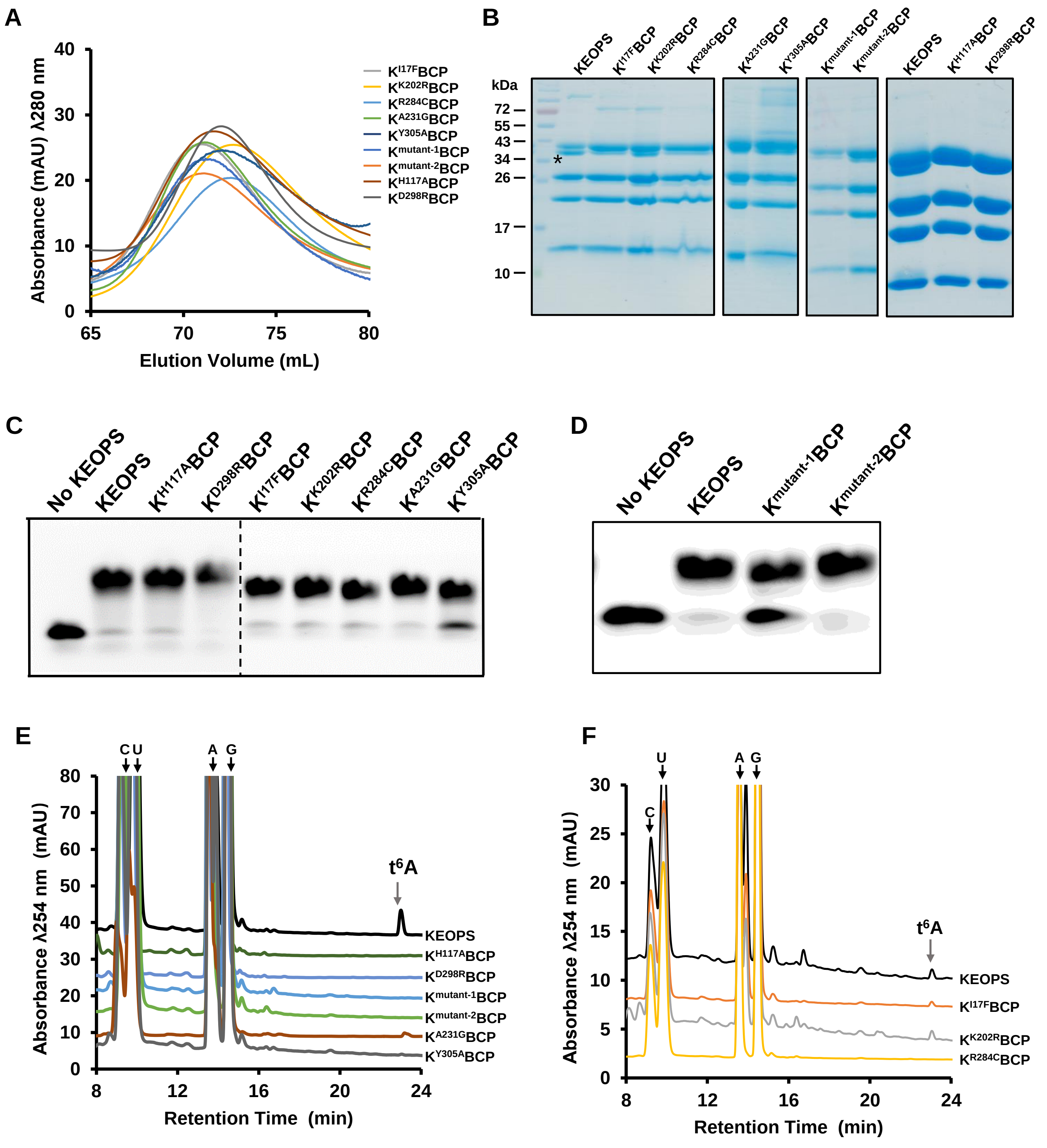

Supplementary Figure 5. Biochemical and functional characterization of KAE1-mutated *At*KEOPS variants. **A.** and **B.** SEC (HiLoad 16/600 Superdex 200, GE Healthcare) profiles (**A**) and SDS–PAGE analysis (**B**) of *At*KEOPS variants bearing mutations in KAE1. mutant-1, Y36R/I37K/T38R/P39R/P40R/G41W/H42D; mutant-2, G43E/F44R/L45K/P46R/R47D/E48K. * indicates KAE1 degradation. **C.** and **D.** EMSA analysis of the interactions between 0.1 μM 5'-6FAM-$tRNA^{Arg}_{CCU}$ and 0.5 μM *At*KEOPS variants (**B**). **E.** and **F.** HPLC chromatograms of nucleosides of the digested *At*$tRNA^{Arg}_{CCU}$ from enzymatic assays using 2 μM *At*YRDC and 2 μM *At*KEOPS variants bearing mutations in KAE1.

Supplementary Figure 6

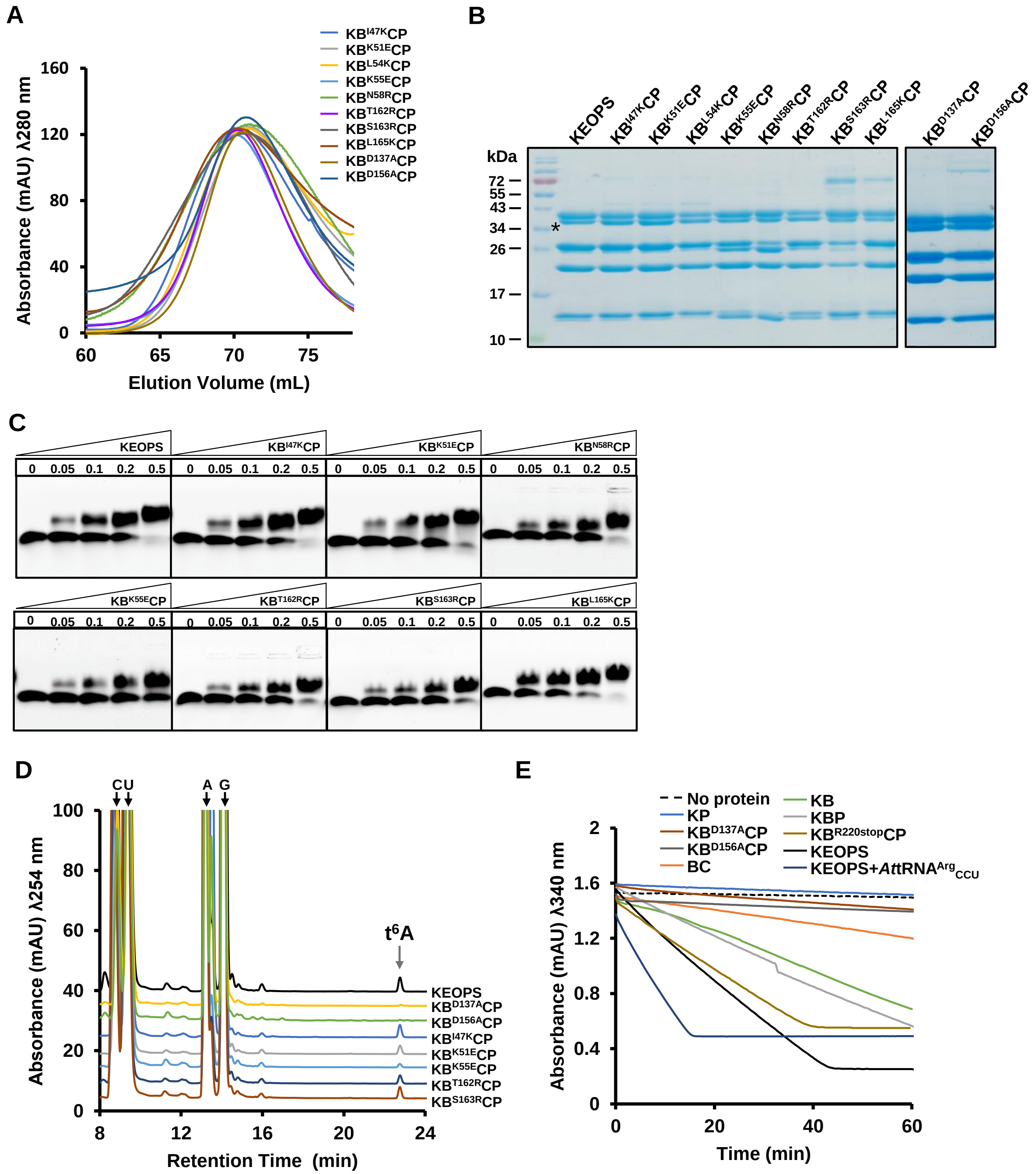

Supplementary Figure 6. Biochemical and functional characterization of BUD32-mutated *At*KEOPS variants. **A.** and **B.** SEC (HiLoad 16/600 Superdex 200, GE Healthcare) profiles (**A**) and SDS–PAGE analysis (**B**) of *At*KEOPS variants bearing mutations in BUD32. * indicates KAE1 degradation. **C.** EMSA analysis of the interactions between 0.1 μM 5'-6FAM-tRNA$^{Arg}_{CCU}$ and 0.05–0.5 μM *At*KEOPS variants (**B**). **D.** HPLC chromatograms of nucleosides of the digested tRNA$^{Arg}_{CCU}$ from enzymatic assays using 2 μM *At*YRDC and 2 μM *At*KEOPS variants bearing mutations in BUD32 (**B**). **E.** NADH-coupled ATPase assay for 5 μM *At*KEOPS complex, subcomplexes and variants in the presence or absence of 10 μM IVT *At*tRNA$^{Arg}_{CCU}$.

Supplementary Figure 7

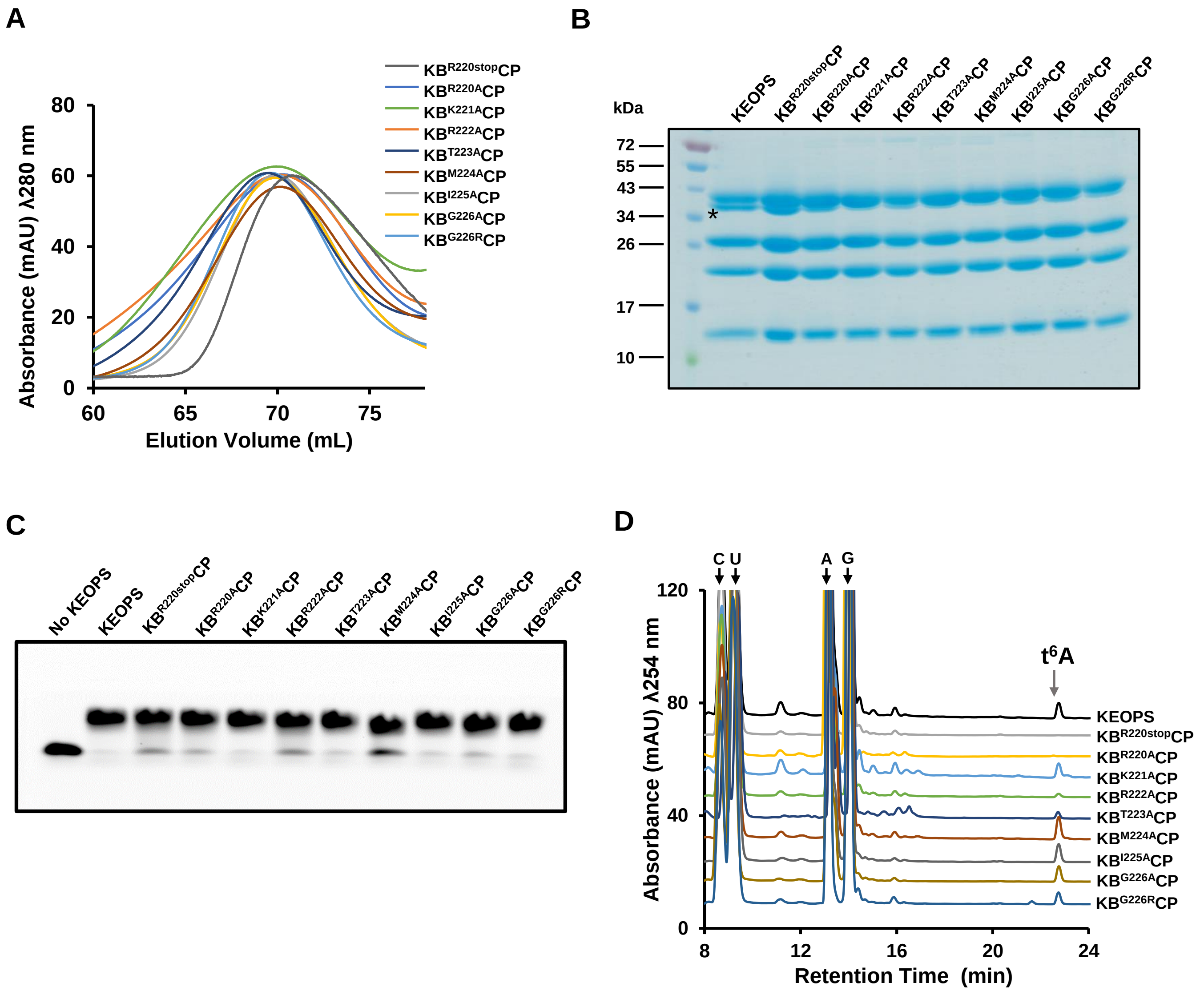

Supplementary Figure 7. Functional characterization of the C-terminal tail of *At*BUD32. **A.** and **B.** SEC (HiLoad 16/600 Superdex 200, GE Healthcare) profiles (**A**) and SDS–PAGE analysis (**B**) of *At*KEOPS variants bearing mutations in the C-terminal tail of BUD32. * indicates KAE1 degradation. **C.** EMSA analysis of the interaction between 0.1 μM 5'-6FAM-tRNA$^{Arg}_{CCU}$ and 0.5 μM *At*KEOPS variants bearing mutations in the C-terminal tail of BUD32 (**B**). **D.** HPLC chromatograms of nucleosides of the digested IVT *At*tRNA$^{Arg}_{CCU}$ from enzymatic assays using 2 μM *At*YRDC and 2 μM *At*KEOPS variants bearing mutations in the C-terminal tail of BUD32 (**B**).
